# Novel electroblowing synthesis of tin dioxide and composite tin dioxide/silicon dioxide submicron fibers for cobalt(ii) uptake†

**DOI:** 10.1039/d1ra01559a

**Published:** 2021-04-23

**Authors:** Johanna Paajanen, Saara Weintraub, Satu Lönnrot, Mikko Heikkilä, Marko Vehkamäki, Marianna Kemell, Timo Hatanpää, Mikko Ritala, Risto Koivula

**Affiliations:** Department of Chemistry, University of Helsinki P.O. Box 55 FI-00014 Finland johanna.paajanen@helsinki.fi

## Abstract

Nanoscale SnO_2_ has many important properties ranging from sorption of metal ions to gas sensing. Using a novel electroblowing method followed by calcination, we synthesized SnO_2_ and composite SnO_2_/SiO_2_ submicron fibers with a Sn : Si molar ratio of 3 : 1. Different calcination temperatures and heating rates produced fibers with varying structures and morphologies. In all the fibers SnO_2_ was detected by XRD indicating the SnO_2_/SiO_2_ fibers to be composite instead of complete mixtures. We studied the Co^2+^ separation ability of the fibers, since ^60^Co is a problematic contaminant in nuclear power plant wastewaters. Both SnO_2_ and SnO_2_/SiO_2_ fibers had an excellent Co^2+^ uptake with their highest uptake/*K*_d_ values being 99.82%/281 000 mL g^−1^ and 99.79%/234 000 mL g^−1^, respectively. Compared to the bare SnO_2_ fibers, the SiO_2_ component improved the elasticity and mechanical strength of the composite fibers which is advantageous in dynamic column operation.

## Abstract 2

Calcination temperature and heating rate markedly affected the structure and morphology of electroblown SnO_2_/SiO_2_ composite fibers. Compared with bare SnO_2_ fibers, the SiO_2_ component improved the mechanical strength but slightly weakened Co^2+^ uptake.

## Graphical abstract

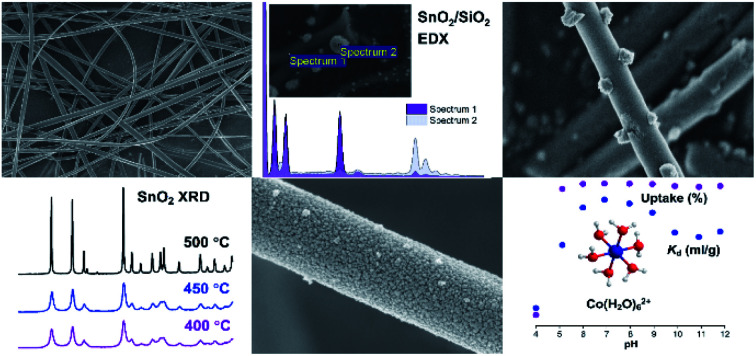

## 1 Introduction

Inorganic materials are interesting as industrial adsorbents due to their good resistance to decomposition at high temperatures and under ionizing radiation and with a wide operating pH range. Furthermore, they are typically much more selective than organic resins for metal ions. The high selectivities stem from their regular, porous and rigid structures that exhibit ion sieve functionality.^1^ There has been research on the sorption characteristics of metal oxides, such as SiO_2_, TiO_2_, ZrO_2_ and SnO_2_ since the mid-20th century.^2^ Among them, tin dioxide nano- and microparticles have shown promising sorption properties for both organic^3^ and inorganic^4–13^ compounds. Nanoscale SnO_2_ has attracted attention as a multifunctional material and it has also been used in transistors,^14^ protective coatings^15^ and gas sensors.^16,17^ Similarly, both bare SnO_2_ and composite SnO_2_/SiO_2_ nanofibers have exhibited promising gas sensing,^18–25^ electrochemical,^26^ optical^27^ and molecular filtration properties.^28^ The large specific surface area and porous structure of fibers provide plenty of contact area for target gases^24,25^ and adsorbing species^28^ and improve the selectivity for adsorbing molecules with different sizes.^28^ The incorporation of an amorphous SiO_2_ component into the SnO_2_ fibers has been shown to improve both the gas sensing properties compared to SnO_2_ nanoparticles^25^ and the structural stability^24^ and mechanical strength^28^ of the fibers.

For removal of heavy metal ions from aqueous solutions, there are plenty of techniques such as chemical precipitation, ion exchange, adsorption, membrane filtration, electrochemical methods and phytoremediation.^29,30^ Adsorption has certain advantages over the other methods including high removal efficiency even at low concentrations, cost-effectiveness, simple design and less production of toxic sludge.^29,31^ Adsorbents comprise both organic and inorganic materials which may be synthetic or of natural origin, such as agricultural by-products. For example, titanium(iv) iodovanadate particles have been utilized for Pb^2+^ and Hg^2+^ separation^32^ and coco-peat biomass for Pb^2+^, Cd^2+^, Cu^2+^ and Ni^2+^ removal.^33^ Inorganic submicron and nanofibers could be excellent adsorbents for a variety of industrial applications. Inorganic fibers have a large specific surface area due to their high surface-to-volume ratio and often form porous structures which can lead to a good adsorption capacity. Zirconium dioxide submicron fibers have performed well in flow-through column operation and shown better mechanical stability, less pressure build-up in the column and faster adsorption kinetics than the corresponding granules.^34^ Submicron and nanofibers can also be an eco-friendly alternative for the purification of nuclear power plant (NPP) wastewaters, since they can markedly reduce the volume of solid radioactive waste that requires a permanent repository.

The most common method to prepare polymeric or inorganic submicron and nanofibers is electrospinning. In this technique, a high voltage is applied to a precursor solution containing a polymer, solvent(s) and for example a metal salt, depending on the desired fiber composition. During the electrospinning process, repulsive electrostatic forces cause the polymer chains to stretch and form fibers that are deposited on a grounded collector. *Via* post-electrospinning calcination, the polymer may be removed and the final fibrous product, typically a metal oxide, is formed. Different precursor solutions and operational parameters allow the control of the properties and morphology of the fibers.^35^ Electrospinning is therefore a simple and cost-efficient method and upscalable for industry.^36–39^ Solution blowing (airbrushing) method in contrast uses pressurized gas to draw fibers from the precursor solution.^40^ Solution blowing offers even 15 times faster production rate than electrospinning^41^ but it tends to result in bundles of aligned fibers whereas electrospinning yields an entanglement of individual, mainly un-aligned fibers.^42,43^ In electroblowing, the fibers are formed by both electrostatic force and air flow and hence the technique combines electrospinning and solution blowing and their advantages. Moreover, electroblowing enables 2.5 times faster solution feeding rate and thus fiber production rate than solution blowing.^41,44^ Compared to electrospinning, the additional air flow in electroblowing permits the use of more viscous precursor solutions^45^ and produces fibers with smaller diameters and fewer beads.^46^ It should be feasible to scale up electroblowing for industry, similarly to electrospinning.

To tackle climate change, global energy production is increasingly based on clean low-carbon power including nuclear power. However, the production of nuclear energy generates radioactive fission and activation products that pose a risk to human health and environment without proper treatment of nuclear waste effluents. Among the activation products, ^60^Co caused by steel corrosion is one of the most hazardous ones due to its rather long half-life of 5.3 years and high gamma decay energies of 1.17 and 1.33 MeV.^47^ Removal of ^60^Co is therefore essential to diminish workers' radiation exposure, to reduce radioactive emissions to aquatic systems and to create safe waste forms for the final disposal. However, the separation of this radionuclide is quite difficult owing to its low concentration in the liquid waste of NPPs (402 Bq L^−1 60^Co in floor drain water, Loviisa NPP, Finland).^48^ The process for its uptake has to be very selective, since generally the liquid waste contains large amounts of inactive metal ions (*e.g.* 37 mg L^−1^ Na^+^ in floor drain water, Loviisa NPP, Finland).^48^

In this research, we prepared SnO_2_ and composite SnO_2_/SiO_2_ submicron fibers by the electroblowing technique and calcination. We also studied the ability of the fibers to remove ^57^Co^2+^ from an aqueous solution. To investigate the effect of SiO_2_ on both the structure and Co^2+^ uptake performance of the SnO_2_ fibers, we added 25 mol% of silicon in the synthesis solution of the composite fibers. The structure and morphology of all the fibers were characterized and their Co^2+^ removal capability was studied. To the best of our knowledge, this is the first report on the electroblowing synthesis of SnO_2_ and SnO_2_/SiO_2_ submicron fibers and on their Co^2+^ uptake. This is also the first report on the uptake of metal ions by fibrous SnO_2_ and SnO_2_/SiO_2_.

## 2 Experimental

### 2.1 Materials

The precursor solutions for the electroblowing experiments were prepared from SnCl_4_·5H_2_O (≥98%, Sigma-Aldrich), tetraethoxysilane (TEOS, Si(C_2_H_5_O)_4_, 98%, Sigma-Aldrich), polyvinylpyrrolidone (PVP, (C_6_H_9_NO)_*n*_, *M*_w_ = 1 300 000, Alfa Aesar), *N*,*N*-dimethylformamide (DMF, C_3_H_7_NO, ≥99.9%, Sigma-Aldrich) ethanol (C_2_H_5_OH, 96 vol%, GPR RECTAPUR) and deionized water. In the uptake experiments ^57^CoCl_2_, Co(NO_3_)_2_·6H_2_O (98%, Sigma-Aldrich), NaNO_3_ (≥99%, VWR Chemicals), NaCl (99.75%, Fisher Chemical), CaCl_2_ (fused, granular, Fisher Chemical), HCl (1 M, Oy FF-Chemicals Ab), NaOH (1 M, Reag. Ph. Eur., VWR Chemicals) and deionized water were used. A stock solution of 5 kBq mL^−1 57^Co^2+^ was prepared by dissolving ^57^CoCl_2_ in 1 mM HCl.

### 2.2 Synthesis of the SnO_2_ and SnO_2_/SiO_2_ fibers

SnO_2_ and SnO_2_/SiO_2_ fibers were synthesized by electroblowing a solution comprising the tin or tin and silicon precursors, PVP and solvents followed by calcination. For the synthesis of SnO_2_ fibers, a certain mass of SnCl_4_·5H_2_O was dissolved in deionized water and then mixed with DMF and 18 wt% PVP/EtOH solution. The mass fractions of the constituents in the solution were 10 wt% for SnCl_4_·5H_2_O, 9 wt% for PVP, 11 wt% for H_2_O, 39 wt% for EtOH and 31 wt% for DMF. For the preparation of SnO_2_/SiO_2_ fibers, SnCl_4_·5H_2_O was dissolved in deionized water and then mixed with DMF, 14 wt% PVP/EtOH solution and TEOS. The mass fractions of the constituents in the solution were 10 wt% for SnCl_4_·5H_2_O, 2 wt% for TEOS, 7 wt% for PVP, 11 wt% for H_2_O, 40 wt% for EtOH and 30 wt% for DMF. The solutions were stirred at room temperature until they became clear and homogeneous.

For the electroblowing, a self-assembled apparatus was used.^49^ In a typical experiment, 12 mL of precursor solution was withdrawn into a syringe and a 27 G (inner diam. 0.21 mm) needle was attached to the syringe. The syringe was placed on a syringe infusion pump (KD Scientific Legato® 101) and the solution feed rate was set to 15 mL h^−1^. This feed rate is 4 to 500 times as high as reported for the electrospinning of SnO_2_ and SnO_2_/SiO_2_ fibers.^21–24,26,50,51^ The needle was pushed through a 3 mm metal adapter of a box enclosing a cylindrical side collector and a planar back collector at 80 cm distance, both being made of a metal wire mesh. The potential difference between the needle and collectors was set to 15 kV with a high voltage power source, and compressed air was delivered through the adapter at a rate of 30 NL min^−1^. The solution jet erupted from the needle tip was deposited as fibers on the grounded collectors and additional drying air was delivered to the box at a rate of 40 NL min^−1^ to enhance solvent evaporation and to control the relative humidity within the box (≤20%). The as-electroblown fibrous mats were detached from the collectors and calcined in an air furnace at 400, 450 and 500 °C for 4 hours with a heating rate of 1 °C min^−1^ in order to remove the polymer and to form the desired ceramic material. The as-electroblown SnO_2_/SiO_2_/PVP fibers were also calcined at 400 °C for 4 hours with heating rates of 5 and 10 °C min^−1^. The yields of SnO_2_ and SnO_2_/SiO_2_ fibers were at best 0.54 and 0.53 g per hour of electroblowing, respectively.

### 2.3 Characterization of the SnO_2_ and SnO_2_/SiO_2_ fibers

Morphology of the fibers was analysed by imaging with secondary electrons (SE) and transmitted electrons (TE) with a Hitachi S-4800 field emission SEM. Prior to the imaging with secondary electrons, the samples were placed on carbon tape and sputter coated with a 4 nm layer of Au/Pd alloy to improve conductivity. Elemental analysis of the fibers including elemental mapping was conducted with an Oxford INCA 350 energy dispersive X-ray spectroscopy (EDX) system connected with the Hitachi S-4800. A Quanta 3D 200i focused ion beam SEM (FIB-SEM) equipped with an Oxford INCA 350 EDX system and an Omniprobe nanomanipulator was used for extracting single SnO_2_/SiO_2_ fibers onto a copper grid for cross-sectional elemental mapping. The average diameters of the fibers and their grains were determined with a Fiji ImageJ software. The crystallinity of the fibers was analysed with a PANalytical X'Pert PRO MPD X-ray diffractometer using Cu Kα radiation and focusing optics. The fiber samples were powdered prior to the analysis. The mean crystallite sizes and their weight ratios were determined from the XRD data by the Rietveld refinement using a MAUD software.^52^ Thermogravimetric analysis (TGA) of the as-electroblown fibers was conducted with a NETZSCH STA 449 F3 Jupiter® system using a heating rate of 10 °C min^−1^ in a temperature range of 25 to 1000 °C in a flow of air (50 mol%) and N_2_ (50 mol%, the purge gas). The specific surface area and porosity of the fibers were measured by N_2_ physisorption at 77 K with a Micromeritics ASAP 2020 Gas sorption analyser.

### 2.4 Co^2+^ uptake studies

#### 2.4.1 Effect of calcination temperature and heating rate

Co^2+^ uptake by the SnO_2_ and SnO_2_/SiO_2_ fibers calcined at different temperatures and with different heating rates was studied at a pH of 6. 20 mg of ground fibers was weighed into 20 mL scintillation vials and 10 mL of 0.01 M NaNO_3_ solution containing 30 Bq mL^−1 57^Co^2+^ was added into the vials. The pH of the solution was adjusted to 6 with a small volume of NaOH. The samples were equilibrated in a constant rotary mixer (50 rpm) for 24 hours after which the equilibrium pH was measured. The samples were phase separated by centrifugation at 4000 rpm (2100*g*) and syringe filtration (Acrodisc LC PVDF, 0.2 μm). The ^57^Co^2+^ uptake efficiency of each sample was determined by pipetting 5 mL of the filtrate into a scintillation vial and measuring the remaining activity with a PerkinElmer Wallac Wizard 3′′ 1480 automatic gamma counter. The ^57^Co^2+^ uptake results are presented by means of distribution coefficient *K*_d_, that describes the distribution of the adsorbate between the adsorbent and solution:1
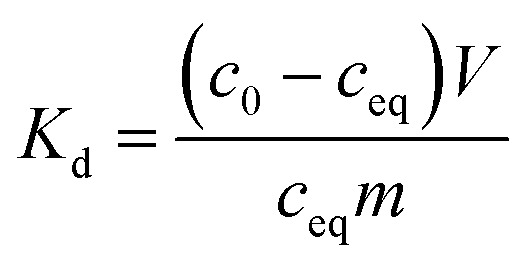
where *c*_0_ (Bq L^−1^) is the initial concentration, *c*_eq_ (Bq L^−1^) is the equilibrium concentration, *V* (mL) is the volume of the solution and *m* (g) is the mass of dry adsorbent. Background activity was subtracted before the calculations. Uncertainty of *K*_d_ was calculated using the error propagation law.

#### 2.4.2 Effect of pH

Co^2+^ uptake by SnO_2_ fibers calcined at 500 °C was investigated in the pH range of 4 to 12. The batch samples were prepared as described above and the pH of the solution was adjusted with a small volume of either HCl or NaOH. The equilibrium pH was measured after the 24 hours of constant rotary mixing. The ^57^Co^2+^ uptake by the fibers was calculated by means of the distribution coefficient *K*_d_ as described above.

#### 2.4.3 Effect of coexisting ions

Selectivity of SnO_2_ fibers towards Co^2+^ in the presence of competing ions Na^+^ and Ca^2+^ was examined. The batch samples were prepared as reported above and the initial pH was adjusted to 6. Four concentrations of the competing ion were used: 0.001, 0.01, 0.1 and 1 M for NaCl, and 0.001, 0.01, 0.1 and 0.5 M for CaCl_2_. The ^57^Co^2+^ removal by the fibers was calculated as the distribution coefficient *K*_d_.

#### 2.4.4 EDX analysis

Elemental analysis of the SnO_2_ and SnO_2_/SiO_2_ fibers after adsorption of Co^2+^ including elemental mapping was conducted. For the analysis, 20 mg of fibers was weighed into 20 mL scintillation vials and 10 mL of 0.01 M NaNO_3_ solution containing 1 mM non-radioactive Co^2+^ (Co(NO_3_)_2_·6H_2_O) was added into the vials. The pH of the solution was adjusted to 6. The samples were equilibrated for 24 h, phase separated and dried in an oven at 70 °C overnight.

## 3 Results and discussion

### 3.1 Electron microscopy and TG analysis of the SnO_2_ and SnO_2_/SiO_2_ fibers

**Fig. 1:**
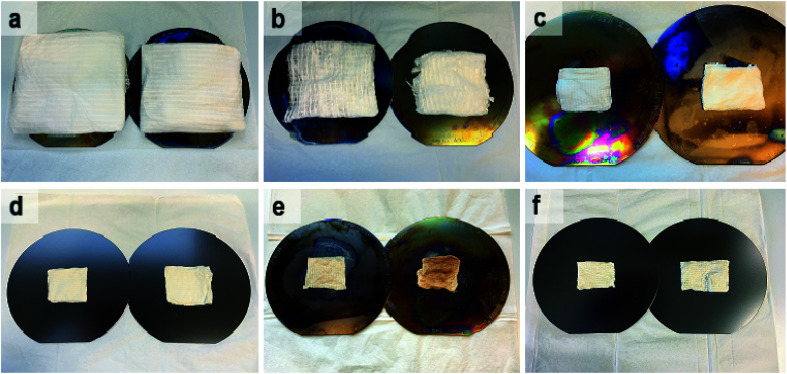
As-electroblown SnO_2_/PVP (a) and SnO_2_/SiO_2_/PVP (b) fibers, SnO_2_ fibers calcined at 400 °C (c) and 500 °C (d) as well as SnO_2_/SiO_2_ composite fibers calcined at 400 °C (e) and 500 °C (f) on 150 mm silicon wafers. In the calcination, a heating rate of 1 °C min^−1^ was used. The calcined fiber mats are shrunken due to the removal of the polymer.

Photographs of as-electroblown SnO_2_/PVP and SnO_2_/SiO_2_/PVP fibers as well as SnO_2_ and SnO_2_/SiO_2_ fibers calcined at 400 and 500 °C are shown in Fig. 1. The calcined fiber mats have shrunk because the polymer has been removed. Except for the SnO_2_/SiO_2_ fibers calcined at 400 °C, the colour of the calcined fibers is white, which implies no major amounts of carbon residues *i.e.* efficient combustion of the polymer. In regard to the SnO_2_/SiO_2_ fibers with a brownish hue, both the lower calcination temperature of 400 °C and the presence of SiO_2_ probably cause incomplete combustion of the PVP. Based on TG analysis,^53^ combustion of the bare PVP fibers is not complete until at 700 °C and hence there may be carbon residues in the calcined fibers although not necessarily to a visible extent. The amorphous SiO_2_ phase in the composite fibers may be more prone to retain the amorphous polymer compared to the crystalline SnO_2_ fibers and SiO_2_ and PVP are known to form hydrogen bonds.^54,55^

**Fig. 2:**
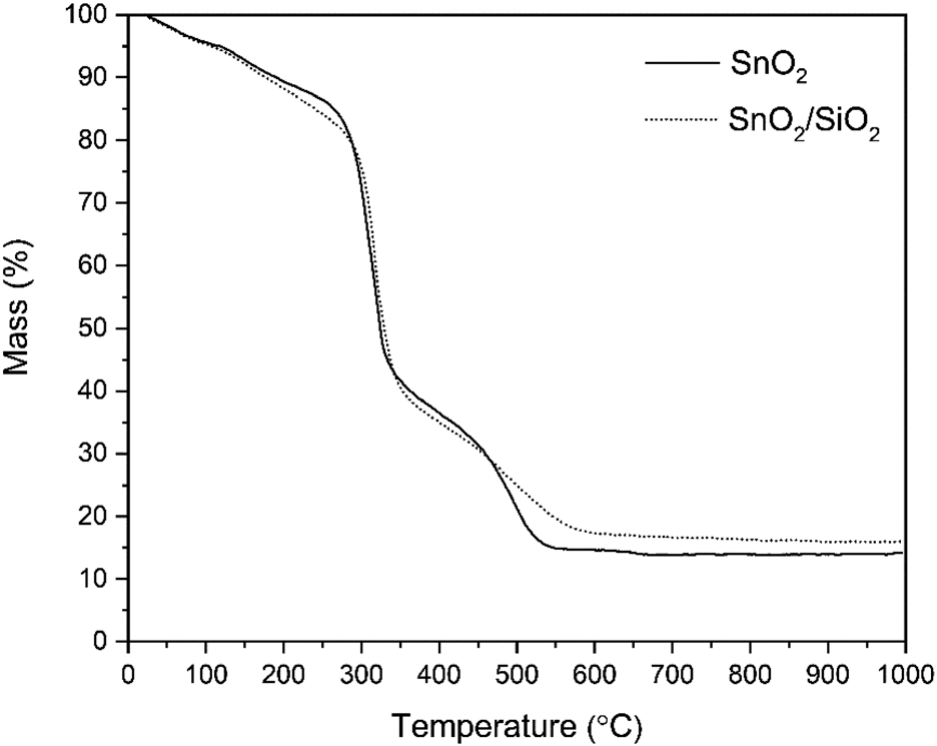
TG curves of as-electroblown SnO_2_/PVP and SnO_2_/SiO_2_/PVP fibers in air (50 mol%) and N_2_ (50 mol%, the purge gas).

According to the TG analysis of the as-electroblown SnO_2_/PVP and SnO_2_/SiO_2_/PVP fibers (Fig. 2), the combustion of the PVP in the SnO_2_/SiO_2_ composite fibers is not complete until at 600 °C whereas it is complete at 500 °C in the case of the bare SnO_2_ fibers. Furthermore, it can be seen that the mass of pure SnO_2_ is *ca.* 15% and the mass of pure SnO_2_/SiO_2_ is *ca.* 20% of the mass of the as-electroblown material. The measured weights of the calcined SnO_2_ and SnO_2_/SiO_2_ fibers are in line with this, also for the lowest calcination temperature of 400 °C. This implies a more effective combustion of the polymer in the calcining furnace which is probably due to a higher oxygen content as the furnace atmosphere comprises air instead of air/N_2_ mixture and a long 4 hour duration of the calcination. Owing to the presence of SiO_2_, the calcined SnO_2_/SiO_2_ composite fiber mats were elastic and could be bent without causing fractures to them. The SnO_2_ fiber mats were more brittle and fractured when bent. The differences in elasticity between the SnO_2_ and SnO_2_/SiO_2_ fibers are demonstrated in the video “Bending experiments with the SnO_2_ and SnO_2_/SiO_2_ fibers”.

**Fig. 3:**
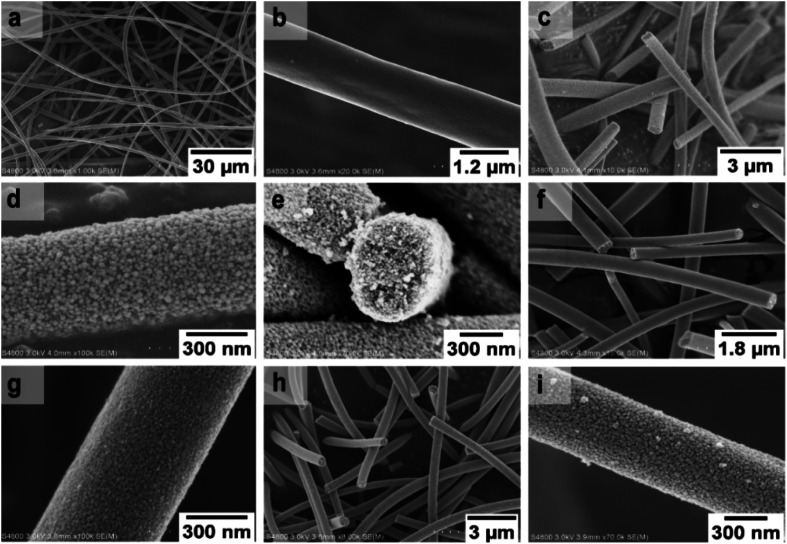
FESEM images at low (a) and high (b) magnification of uncalcined SnO_2_/PVP fibers and SnO_2_ fibers calcined at 400 °C (c to e), 450 °C (f and g) and 500 °C (h and i).

**Fig. 4:**
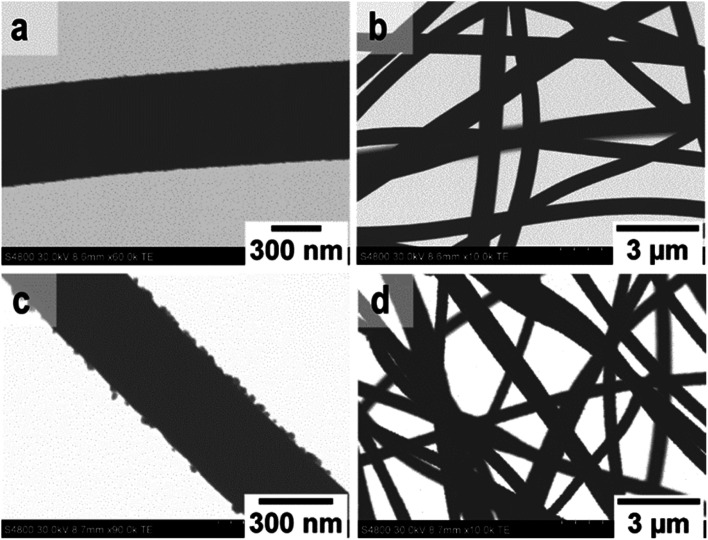
TE images at low and high magnification of SnO_2_ fibers (a and b) and SnO_2_/SiO_2_ composite fibers (c and d) calcined at 500 °C.

FESEM images of the uncalcined SnO_2_/PVP fibers and SnO_2_ fibers calcined at 400, 450 and 500 °C are presented in Fig. 3. The average diameter of the uncalcined fibers containing the polymer was 1.5 μm whereas the average diameter of the calcined fibers was 560 nm. The morphology of the calcined SnO_2_ fibers was similar regardless of the calcination temperature. The fibers seemed to have a uniform structure that consisted of roundish grains approximately 20 nm in diameter, although there was some variation between individual grains. A similar granular structure has also been reported for both dense^21,23,26,50^ and hollow^22,56^ electrospun SnO_2_ nanofibers. The dense character of the fibers in the current study is proved by the FESEM image of a fiber cross-section (Fig. 3e) and was verified by imaging with transmitted electrons (Fig. 4a and b).

**Fig. 5:**
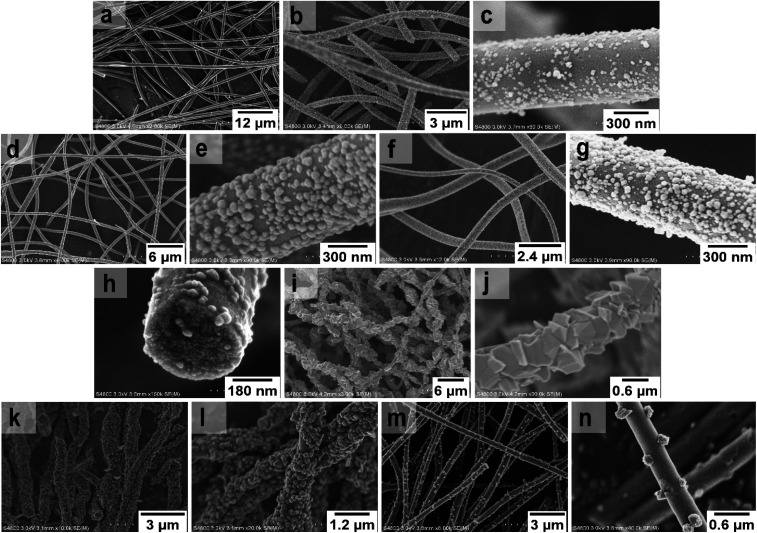
FESEM images of uncalcined SnO_2_/SiO_2_/PVP fibers (a), and images at low and high magnification of SnO_2_/SiO_2_ composite fibers calcined at 400 °C (b and c), 450 °C (d and e) and 500 °C (f–j) with a heating rate of 1 °C min^−1^ as well as SnO_2_/SiO_2_ composite fibers calcined at 400 °C with heating rates of 5 °C min^−1^ (k and l) and 10 °C min^−1^ (m and n).

FESEM images of the uncalcined SnO_2_/SiO_2_/PVP fibers and SnO_2_/SiO_2_ fibers calcined at 400, 450 and 500 °C with a heating rate of 1 °C min^−1^ as well as at 400 °C with heating rates of 5 and 10 °C min^−1^ are presented in Fig. 5. The average diameters of the uncalcined and calcined fibers were 1.2 μm and 580 nm, respectively. Irrespective of the calcination temperature, the appearance of the fibers calcined with a heating rate of 1 °C min^−1^ was the same (Fig. 5b–h). The fibers seemed to have a smooth core that was rather sparsely covered with roundish grains approximately 30 nm in diameter. A rather similar structure comprising a smooth core and granular surface has been reported previously for SnO_2_/SiO_2_ nanofibers, although with a higher proportion of Si from 50 to 83 mol% compared to ours of 25 mol%.^24^ As for the fibers calcined with the faster heating rates, they also had a smooth core sparsely covered with grains that were quite angular in shape and rather large (Fig. 5k–n). The average diameter of the grains was *ca.* 200 nm in the fibers calcined with heating rates of 5 and 10 °C min^−1^, although there was some variation between individual fibers. Among the fibers calcined at 500 °C with the slow heating rate of 1 °C min^−1^, there were also some fibers with a special morphology with the fiber core covered with large, irregular grains of 500 nm to 1 μm in diameter (Fig. 5i and j). FESEM (Fig. 5h) and TE imaging (Fig. 4c and d) confirmed that the structure of the SnO_2_/SiO_2_ composite fibers was dense like that of the bare SnO_2_ fibers.

### 3.2 EDX analysis of the SnO_2_ and SnO_2_/SiO_2_ fibers

EDX spectra of the SnO_2_ fibers are shown in Fig. S1 and S3.† As for the SnO_2_/SiO_2_ composite fibers, the quantitative Sn : Si EDX results were 77 at% : 23 at% and 80 at% : 20 at% for the fibers calcined at 400 and 500 °C, respectively (Fig. S2 and S4†). Considering possible variation in sample homogeneity, the results are as expected and indicate a successful synthesis. Owing to the difficult quantification of light elements with EDX and because detected carbon may also originate in the environment, the amount of residual carbon in the fibers could not be reliably determined.

**Fig. 6:**
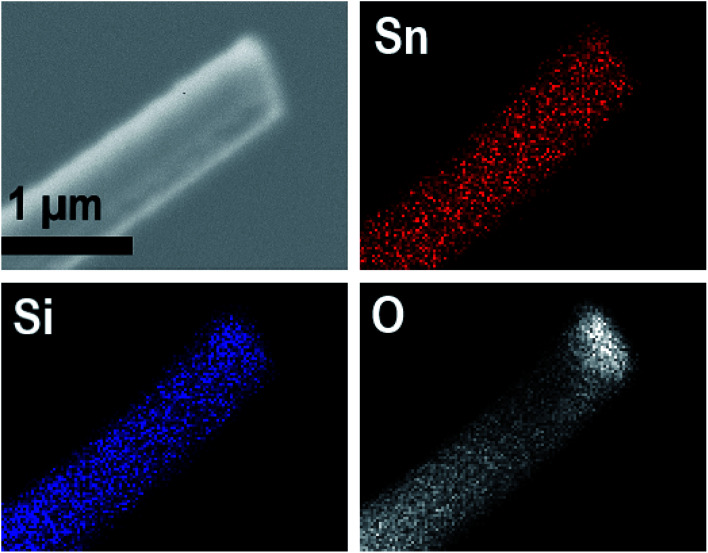
EDX elemental maps of a single SnO_2_/SiO_2_ composite fiber calcined at 500 °C.

**Fig. 7:**
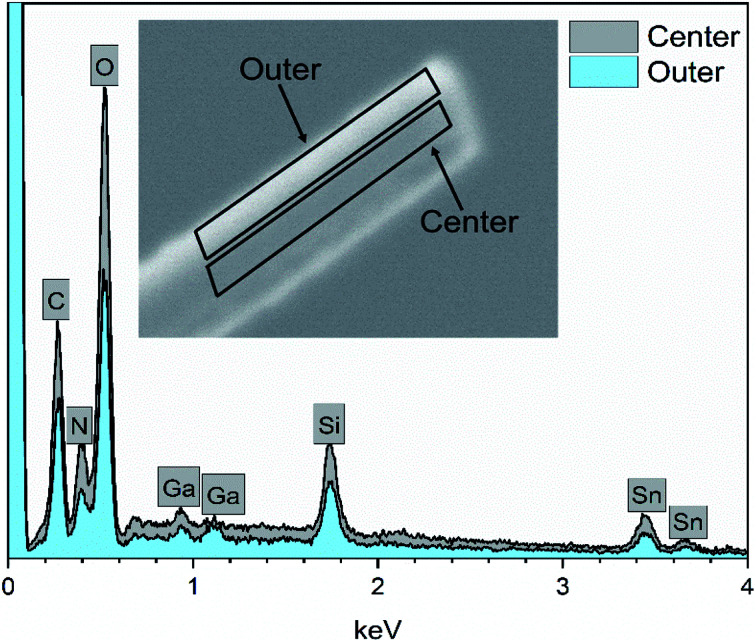
EDX spectra of a single SnO_2_/SiO_2_ composite fiber calcined at 500 °C measured at the longitudinal cross-section surface. Ga peaks are due to the ion beam used for fiber milling.

**Fig. 8:**
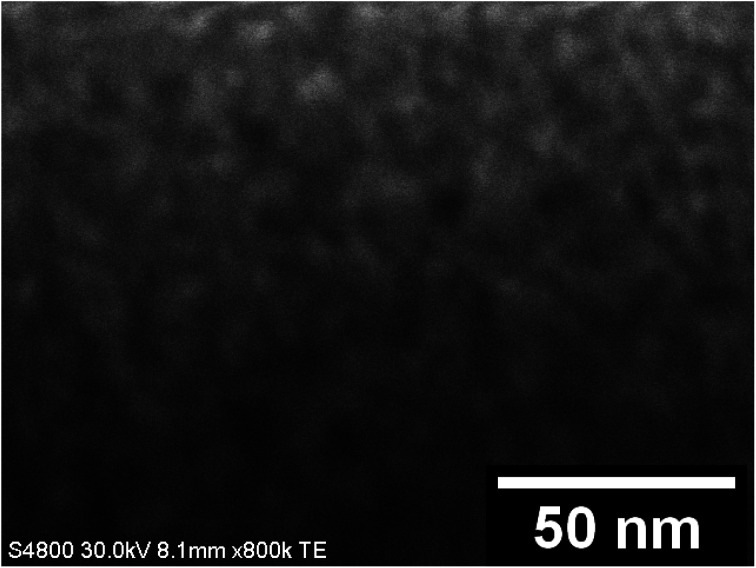
A TE image of the inner section of a single SnO_2_/SiO_2_ composite fiber calcined at 500 °C.

EDX elemental maps of a single SnO_2_/SiO_2_ fiber calcined at 500 °C are shown in Fig. 6. EDX spectra recorded from both inner and outer section of the smooth part of the fiber are presented in Fig. 7. A TE image of a thin inner section of the fiber is shown in Fig. 8. For the EDX measurement, a portion of the fiber was FIB milled away to expose a flat longitudinal cross-section surface from which the spectra were recorded. For the TE imaging, equal longitudinal portions from both sides of the fiber were FIB milled away leaving a thin slab in the middle of which the imaging was done. The elemental maps prove the presence of Sn, Si and O in the fiber but on the basis of them it is difficult to tell any difference between the distribution or the concentration of Sn and Si in the fiber. The EDX spectra of the center and outer smooth parts of the fiber are quite identical and they both show the presence of Sn. This implies that the core of the SnO_2_/SiO_2_ composite fibers in this study is different from the single-phase amorphous SiO_2_ core reported for the SnO_2_/SiO_2_ composite fibers with a higher proportion of Si.^24^ The TE image from inside the fiber supports this as it suggests that the inner part of the fiber is not homogeneous but consists of distinct nanoscale domains, possibly small SnO_2_ grains embedded in a SiO_2_ matrix. Previously it has been proved that more than *ca.* 1 mol% of SnO_2_ cannot be dissolved in SiO_2_ but SnO_2_ forms crystalline nanoclusters dispersed in amorphous SiO_2_.^57,58^

**Fig. 9:**
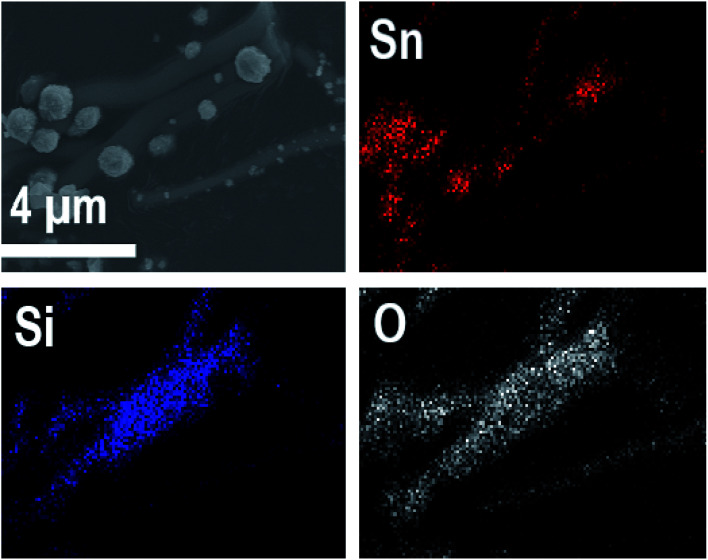
EDX elemental maps of SnO_2_/SiO_2_ composite fibers calcined at 400 °C with a heating rate of 10 °C min^−1^.

**Fig. 10:**
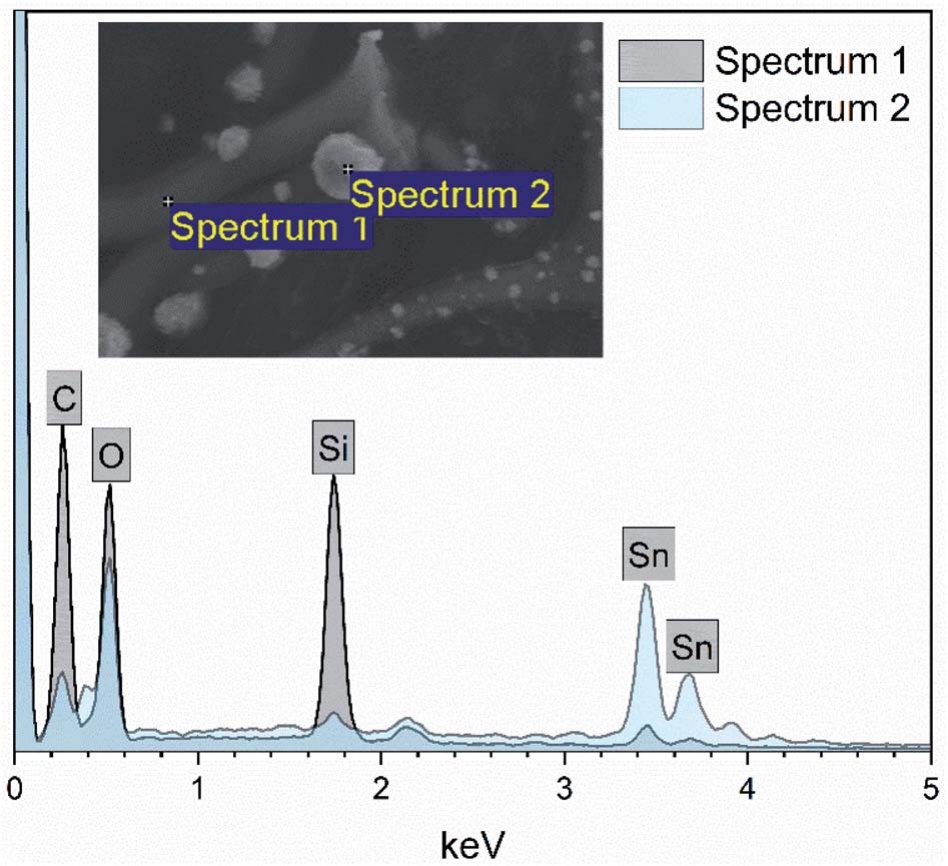
EDX spectra measured at the smooth part (spectrum 1) and surface grain (spectrum 2) of a single SnO_2_/SiO_2_ composite fiber calcined at 400 °C with a heating rate of 10 °C min^−1^.

EDX elemental maps of the SnO_2_/SiO_2_ composite fibers calcined at 400 °C with a heating rate of 10 °C min^−1^ are presented in Fig. 9. As can be seen, Sn is more concentrated at the sites where the large grains are located while Si is more concentrated at the smooth part of the fibers. This implies that the large grains on the surface of the fibers consist primarily of SnO_2_. More evidence is provided by EDX spectra (Fig. 10) recorded at the smooth part (spectrum 1) and at a surface grain (spectrum 2) of the fibers. In the spectrum recorded at the grain there are strong signals for Sn and a very weak signal for Si while in the spectrum recorded at the smooth part the case is the opposite, *i.e.* the signal for Si is very intense and the signals for Sn are quite low.

### 3.3 Crystal structure of the SnO_2_ and SnO_2_/SiO_2_ fibers

**Fig. 11:**
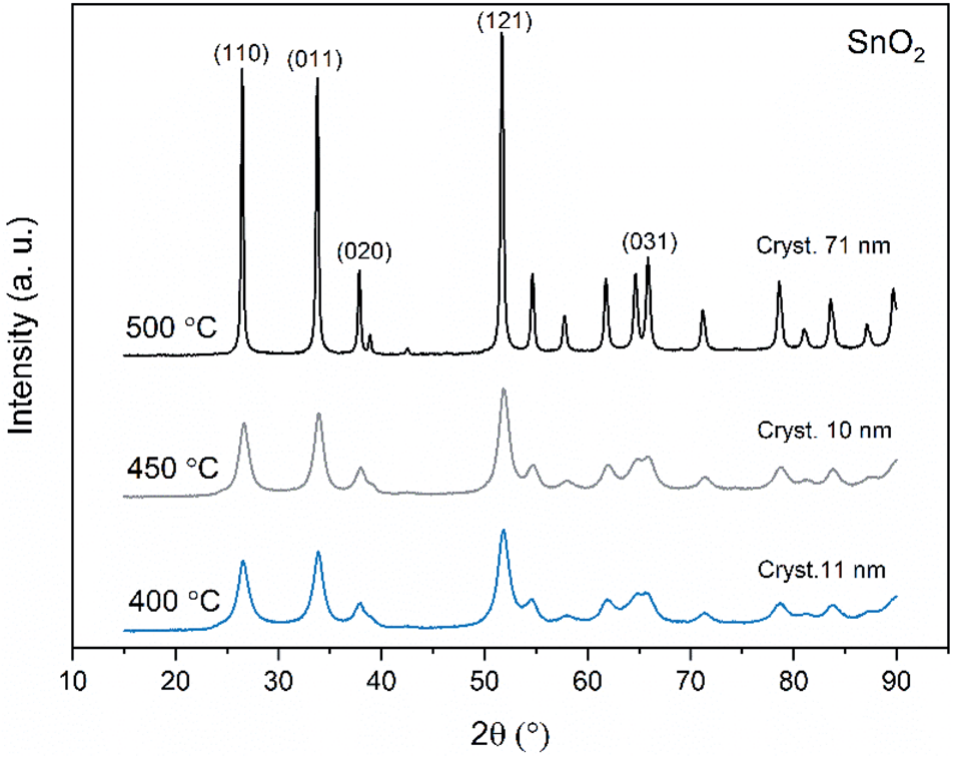
X-ray diffraction patterns of the SnO_2_ fibers calcined at 400, 450 and 500 °C. Cryst. refers to average crystallite size in this and Fig. 12 and 13.

**Fig. 12:**
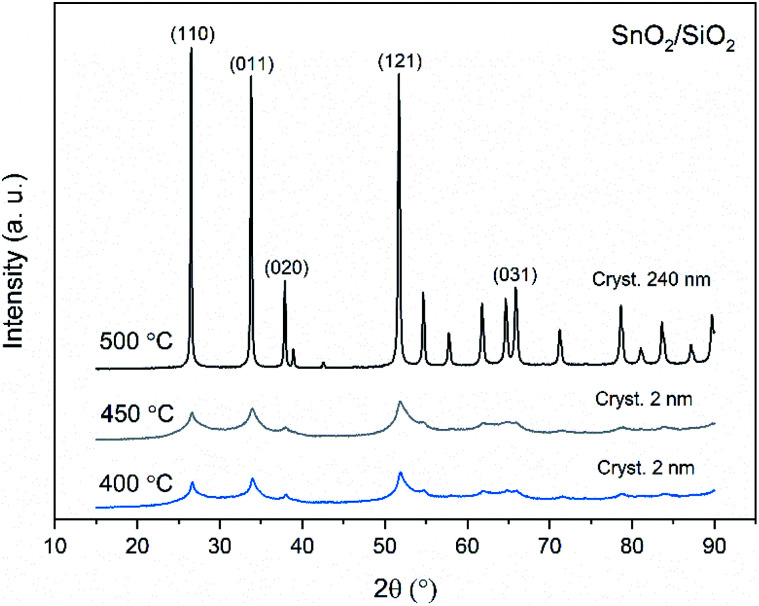
X-ray diffraction patterns of the SnO_2_/SiO_2_ fibers calcined at 400, 450 and 500 °C with a heating rate of 1 °C min^−1^.

**Fig. 13:**
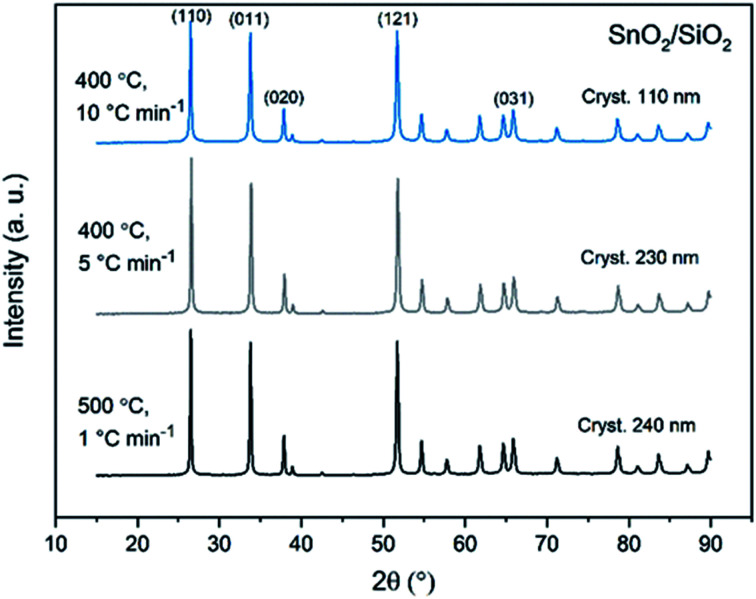
X-ray diffraction patterns of the SnO_2_/SiO_2_ fibers calcined at 500 °C with a heating rate of 1 °C min^−1^ and at 400 °C with heating rates of 5 and 10 °C min^−1^.

X-ray diffraction patterns of both bare SnO_2_ and composite SnO_2_/SiO_2_ fibers are presented in Fig. 11–13. All the fibers irrespective of the calcination temperature or heating rate have the tetragonal rutile structure. The presence of SiO_2_ doesn't seem to affect the crystalline phase nor the lattice parameters compared to bare SnO_2_ fibers but instead it affects the crystallite size. Interestingly, in both SnO_2_ and SnO_2_/SiO_2_ fibers there were crystallites of two different sizes for which the weight ratios were determined (Table 1). Refining the XRD data with a single crystallite size led to unsatisfactory results while clear improvement was achieved by using two different crystallite sizes with varying weight ratios. This was pronounced with the composite fibers. However, the bimodal size distribution may not be sufficient and a more complex distribution is probable, but from the perspective of this study it is adequate to show that there are at least two different crystallite sizes present. The fraction of one size was always much higher than that of the other size, ranging from 63 to 93 wt%. In all the fibers the larger crystallites were predominant over the smaller ones, except for the composite fibers calcined at 400 and 450 °C with a heating rate of 1 °C min^−1^ (Fig. 5b–e). The sizes of the proportionally larger crystallites varied between 10 and 243 nm; the fibers calcined at 400 and 450 °C with slow heating possessed the smallest of them and the fibers calcined at 500 °C or at 400 °C with fast heating the largest of them. The sizes of the proportionally smaller crystallites varied from 2 to 15 nm and also here the size followed calcination temperature and heating rate: the fibers calcined at 400 and 450 °C with slow heating possessed the smallest of them and the fibers calcined at 500 °C or at 400 °C with fast heating the largest of them. Rietveld refined X-ray diffractograms of SnO_2_ and SnO_2_/SiO_2_ fibers calcined at 500 °C are presented in Fig. S13 and S14.†

**Table 1:** Average crystallite sizes of the SnO_2_ and SnO_2_/SiO_2_ fibers and weight ratios of small (low crystallinity) and large (high crystallinity) crystallites. Calc. param. refer to calcination parameters, *i.e.* calcination temperature and heating rate. The calcination duration was 4 h

Fibers	Calc. param.	Crystallinity	Av. cryst. size (nm)	Wt%
SnO_2_	400 °C, 1 °C min^−1^	High/low	11/3	63/37
SnO_2_	450 °C, 1 °C min^−1^	High/low	10/2	93/7
SnO_2_	500 °C, 1 °C min^−1^	High/low	71/12	87/13
SnO_2_/SiO_2_	400 °C, 1 °C min^−1^	Low/high	2/14	83/17
SnO_2_/SiO_2_	450 °C, 1 °C min^−1^	Low/high	2/11	80/20
SnO_2_/SiO_2_	500 °C, 1 °C min^−1^	High/low	243/11	79/21
SnO_2_/SiO_2_	400 °C, 5 °C min^−1^	High/low	230/15	74/26
SnO_2_/SiO_2_	400 °C, 10 °C min^−1^	High/low	111/9	74/26

The crystallites in the SnO_2_ fibers calcined at 400 and 450 °C are in average 11 and 10 nm in size, respectively, whereas in the corresponding SnO_2_/SiO_2_ fibers they are only 2 nm in size. The silica in the composite fibers probably acts as a crystal growth inhibitor, as is also the case with SnO_2_ nanoparticles embedded in a SiO_2_ network.^59^ It should be noted though that the refinement results of the broad bumps in this data (Fig. 12) leading to below 10 nm sized crystallites represent rather the lack of long range order than an actual precise size, since severe overlapping of the peaks showing poor crystallinity undermines the accuracy of the results. In both the bare and composite fibers, the average crystallite size increases substantially as the calcination temperature is increased and in the composite fibers the increase is quite steep (Table 1). It is known that the crystallite size of ceramic submicron fibers depends on the calcination temperature.^44^ In the bare SnO_2_ fibers, the crystallite growth is probably accompanied with a grain growth at the higher temperatures^21^ although no prominent difference in grain size between the fibers calcined at 400, 450 and 500 °C was observed on the basis of the FESEM analysis (Fig. 3). As for the composite fibers calcined at 500 °C with a heating rate of 1 °C min^−1^ and at 400 °C with heating rates of 5 and 10 °C min^−1^, both the high calcination temperature and fast heating rates could explain the large crystallite sizes of 243, 230 and 111 nm, respectively (Table 1). It is likely that the large, irregular grains on the fibers (Fig. 5i–n) consist mainly of SnO_2_ that is known to form various shapes in nanoscale.^50,60^ High calcination temperatures and fast heating rates may influence both the size and shape of crystals,^61^ which is reflected in the morphology of grains comprising the crystals.

The presence of crystallites of two different sizes in the SnO_2_/SiO_2_ composite fibers is reasonable in the light of the electron microscopy and EDX analysis results. Based on EDX analysis, both Sn and Si are present in the core of the composite fibers but the surface grains consist primarily of SnO_2_ (Fig. 7, 9 and 10). It is seen in the TEM image (Fig. 8) that the core of the fibers comprises different nanoscale domains probably of SnO_2_ and SiO_2_. As discussed, SiO_2_ in the core of the fibers presumably prevents SnO_2_ crystallite growth. This might cause the presence of small SnO_2_ crystallites and grains in the core of the fibers. The SnO_2_ crystallites can grow more freely on the surface of the fibers resulting in larger crystallite and grain sizes. For the SnO_2_/SiO_2_ fibers calcined at 400 and 450 °C with a heating rate of 1 °C min^−1^ the relatively high amount of small 2 nm crystallites (83 and 80 wt%, respectively, Table 1) and the moderate size of the larger crystallites (14 and 11 nm, respectively, Table 1) are probably due to the low calcination temperature and slow heating rate. As regards the bare SnO_2_ fibers, the presence of the smaller crystallites (3, 2 and 12 nm for the fibers calcined at 400, 450 and 500 °C, respectively, Table 1) might be at least partly due to some PVP residues hindering crystal growth. This may especially be the case for the fibers calcined at the lowest temperature of 400 °C for which the fraction of the small crystallites, 37 wt%, is the highest of the SnO_2_ fibers. The average crystallite sizes of 11, 10 and 12 nm (SnO_2_, Table 1) as well as 14, 11 and 11 nm (SnO_2_/SiO_2_, Table 1) for the fibers calcined at 400, 450 and 500 °C with slow heating, respectively, coincide with some SnO_2_ grain sizes seen in the FESEM images (Fig. 3d, e, g, i and 5c, e, g). Previously it has been found that the crystal size of granular polycrystalline SnO_2_ nanofibers is approximately the same as the size of the SnO_2_ grains meaning that each SnO_2_ grain is possibly a single crystal.^26^ Perhaps this is the case for some SnO_2_ grains in the fibers of this study as well.

### 3.4 Specific surface area, pore volume and pore size analysis of the SnO_2_ fibers

Specific surface area of the SnO_2_ fibers calcined at 500 °C was analysed by the Brunauer–Emmett–Teller (BET) method and total pore volume, pore size distribution and average pore diameter by the Barrett–Joyner–Halenda (BJH) method using nitrogen gas adsorption and desorption. The specific surface area, total pore volume and average pore size of the fibers were 41 m^2^ g^−1^, 0.15 cm^3^ g^−1^ and 10 nm, respectively. The surface area of the fibers is somewhat higher than that found in literature for both dense^51^ and hollow^56,62^ electrospun SnO_2_ fibers calcined at 500 or 600 °C, 6.9 to 36 m^2^ g^−1^. The same is true of the total pore volume, as a smaller pore volume of 0.079 cm^3^ g^−1^ has been reported by Mudra *et al.*^62^ while a larger average pore size of 16 nm has been reported by Xia *et al.*^56^ The surface area of the SnO_2_ fibers is quite high considering the large 71 nm crystallites, as large crystallites commonly result in a small surface area. For ZrO_2_ fibers of almost the same diameter (570 nm) crystallites of 9 and 63 nm produced surface areas of 14 and 1.7 m^2^ g^−1^, respectively.^44^ The large surface area of the SnO_2_ fibers of this study can probably be explained by the granular and porous structure. The average pore size of 10 nm seems to match quite well with the occasional interstices between the grains of the fibers (Fig. 3d, e, g and i). N_2_ adsorption and desorption isotherms and pore size distribution of the SnO_2_ fibers are presented in Fig. S15 and S16,† respectively.

### 3.5 Co^2+^ uptake by the SnO_2_ and SnO_2_/SiO_2_ fibers

The adsorption ability of electroblown submicron fibers is greatly affected by their crystal structure and size, morphology and the amount of possible polymer residues. These characteristics, in turn, depend on the calcination temperature.^44^ Therefore, we investigated the effects of calcination temperature and heating rate on the Co^2+^ uptake by the SnO_2_ and SnO_2_/SiO_2_ fibers. As revealed by Table 2, all the fibers have a good Co^2+^ uptake performance with an average uptake of 99.71% and 99.53% for SnO_2_ and SnO_2_/SiO_2_ fibers, respectively. However, there were some differences between the fibers. As seen from the *K*_d_ values, the bare SnO_2_ fibers have somewhat better Co^2+^ uptake than the SnO_2_/SiO_2_ composite fibers. The SiO_2_ in the composite fibers seems to impair their Co^2+^ uptake, although chemisorption of Co^2+^ on SiO_2_ is known.^63^ In this study, however, the crystalline SnO_2_ appears to be the major adsorbent. The high surface area of the SnO_2_ fibers is likely to enhance the adsorption even more as it should provide plenty of adsorption sites. EDX elemental maps and spectra of the SnO_2_ and SnO_2_/SiO_2_ fibers calcined at 400 and 500 °C with a heating rate of 1 °C min^−1^ after adsorption of Co^2+^ are shown in Fig. S5–S12.†

**Table 2:** ^57^Co^2+^ uptake by the SnO_2_ and SnO_2_/SiO_2_ fibers in 0.01 M NaNO_3_ at pH 6.0. Calc. param. refer to calcination parameters, *i.e.* calcination temperature and heating rate. The calcination duration was 4 h

Fibers	Calc. param.	Uptake (%)	*K*_d_ (mL g^−1^)
SnO_2_	400 °C, 1 °C min^−1^	99.51 ± 0.02	173 000 ± 3000
SnO_2_	450 °C, 1 °C min^−1^	99.81 ± 0.02	272 000 ± 4000
SnO_2_	500 °C, 1 °C min^−1^	99.82 ± 0.01	281 000 ± 5000
SnO_2_/SiO_2_	400 °C, 1 °C min^−1^	99.69 ± 0.02	163 000 ± 3000
SnO_2_/SiO_2_	450 °C, 1 °C min^−1^	99.18 ± 0.02	58 100 ± 1000
SnO_2_/SiO_2_	500 °C, 1 °C min^−1^	99.28 ± 0.02	69 700 ± 1300
SnO_2_/SiO_2_	400 °C, 5 °C min^−1^	99.70 ± 0.02	168 000 ± 3000
SnO_2_/SiO_2_	400 °C, 10 °C min^−1^	99.79 ± 0.02	234 000 ± 4000

**Fig. 14:**
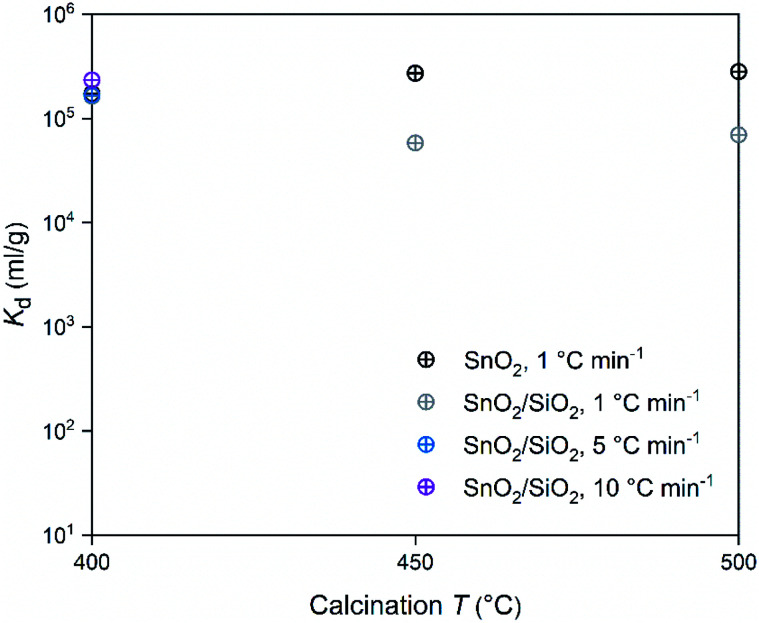
Distribution coefficient of 30 Bq mL^−1 57^Co^2+^ on SnO_2_ and SnO_2_/SiO_2_ fibers as a function of calcination temperature and heating rate in 0.01 M NaNO_3_ at pH 6.0 (2 g L^−1^ SnO_2_ or SnO_2_/SiO_2_).

In regard to the SnO_2_ and SnO_2_/SiO_2_ fibers calcined with a heating rate of 1 °C min^−1^, the calcination temperature had a different effect on their Co^2+^ uptake performance. Calcination temperatures of 500 and 400 °C produced the best adsorption performance for SnO_2_ and SnO_2_/SiO_2_ fibers, respectively (Table 2 and Fig. 14). Lower calcination temperature tends to produce smaller crystallites and thus increase the surface area of the material, which, in turn, improves its adsorption properties.^44^ Therefore it is surprising that the SnO_2_ fibers calcined at 500 °C possessing 71 nm crystallites perform better than the SnO_2_ fibers calcined at 400 and 450 °C possessing 11 and 10 nm crystallites, respectively. One possible reason is that the lower calcination temperatures leave some PVP residues in the material blocking some of the adsorption sites. However, despite their different crystallite size, the *K*_d_ value of the SnO_2_ fibers calcined at 450 °C is almost as high as that of the fibers calcined at 500 °C. The crystallite size doesn't seem to be a determining factor in the Co^2+^ uptake performance of the SnO_2_ fibers.

In the SnO_2_/SiO_2_ fibers calcined with a heating rate of 1 °C min^−1^ the Co^2+^ uptake correlated better with the crystallite size, since the fibers calcined at 400 °C and with the smallest 2 nm crystallites performed the best (Table 2 and Fig. 14). It should be noted that although the fibers calcined at 450 °C also possessed 2 nm crystallites, their sorption and *K*_d_ values were lower. One reason for the weaker Co^2+^ uptake performance of the SnO_2_/SiO_2_ fibers calcined at 450 and 500 °C with a heating rate of 1 °C min^−1^ could be that due to the higher calcination temperature, they have fewer surface hydroxyl groups that are assumed to play a significant role in the uptake process. As for the fibers calcined at 500 °C, the peculiar morphology of some of the fibers (Fig. 5i and j) and large crystallite size of 243 nm that decreases the surface area, may also impair their Co^2+^ uptake performance. The SnO_2_/SiO_2_ fibers calcined at 400 °C with the fast heating rates of 5 and 10 °C min^−1^ exhibited excellent Co^2+^ uptake despite their large crystallite sizes. This might be explained by the roundish shape and quite sparse distribution of their SnO_2_ grains (Fig. 5k–n) compared to the angular and rather tightly packed SnO_2_ grains of the fibers calcined at 500 °C (Fig. 5i and j). The roundish and sparsely distributed surface SnO_2_ grains might be better accessible to adsorbing ions in an aqueous solution. Moreover, the lower target temperature of 400 °C may cause more hydroxyl groups remaining on the surface of the fibers enhancing the uptake of Co^2+^.

**Fig. 15:**
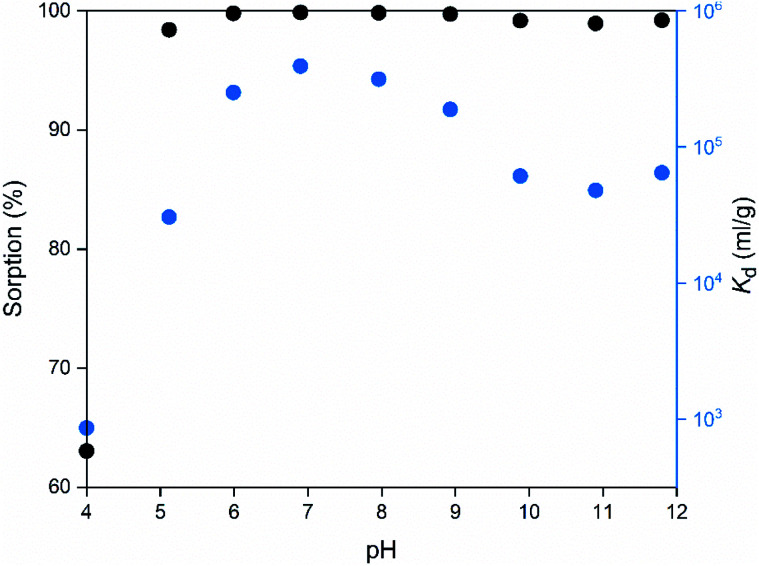
Sorption and distribution coefficient of 30 Bq mL^−1 57^Co^2+^ on SnO_2_ fibers calcined at 500 °C as a function of pH in 0.01 M NaNO_3_ (2 g L^−1^ SnO_2_).

The pH of the solution may have a major impact on the uptake properties of a material because it affects both the speciation of the adsorbate and the surface charge of the adsorbent. As for Co^2+^, it exists as Co(H_2_O)_6_^2+^ in the pH range of 2 to 9.^64^ It can be inferred from the *K*_d_ values (Fig. 15), that the SnO_2_ fibers adsorb Co^2+^ the best in the neutral to mildly basic pH region, from pH 6 to 9, and they reach the highest *K*_d_ value at the pH of 7. This is in accordance with previous research^7^ and promising for the use of the fibers in purification of NPP waste waters, since the pH of the primary coolant water in NPPs is about 7. The point of zero charge (pH_pzc_)of SnO_2_ lies at a pH of 4,^7^ and above this pH the surface charge of pure SnO_2_ is negative. The Co^2+^ uptake by the rutile structured SnO_2_ is evidently based on electrostatic forces^65^ which explains the good uptake in the neutral to mildly basic pH region where the charges of the Co^2+^ species and the surface of the SnO_2_ fibers are the opposite. The adsorption of Co^2+^ on hydrous SnO_2_ most probably occurs *via* substitution of H^+^ ions of the surface water molecules or hydroxyl groups by Co^2+^ ions.^4–6,66^ An assumed ion exchange reaction between hydrous SnO_2_ and Co^2+^ is illustrated in Fig. 17.^5^

**Fig. 16:**
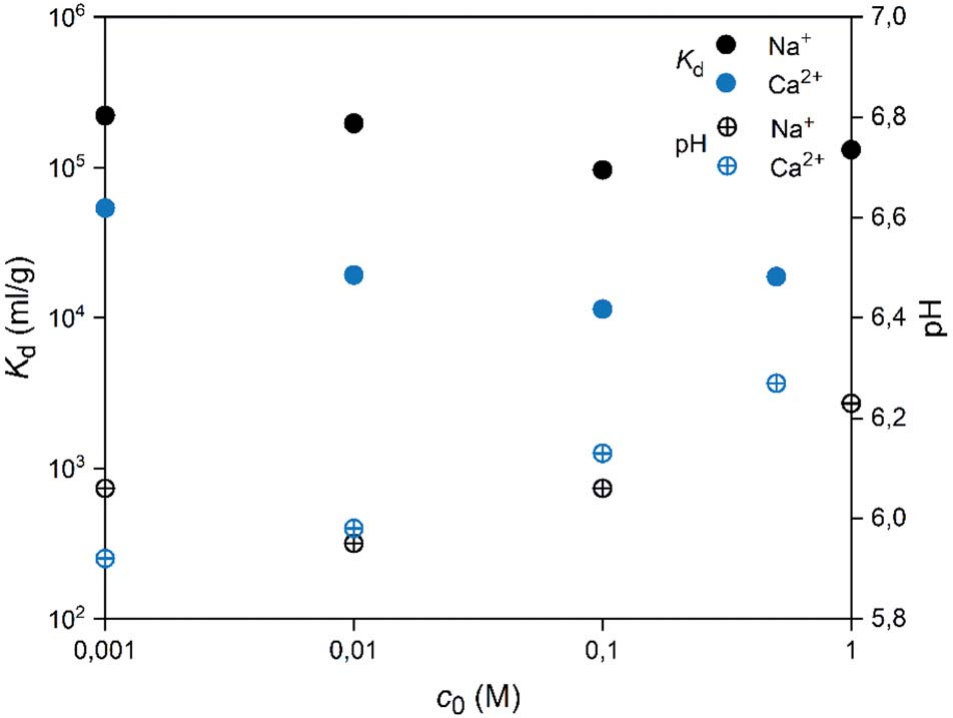
Effects of concentrations of competing ions Na^+^ or Ca^2+^ on the uptake of 30 Bq mL^−1 57^Co^2+^ by SnO_2_ fibers calcined at 500 °C. The initial pH was 6 and the pH at the end of the experiment is shown (2 g L^−1^ SnO_2_).

**Fig. 17:**
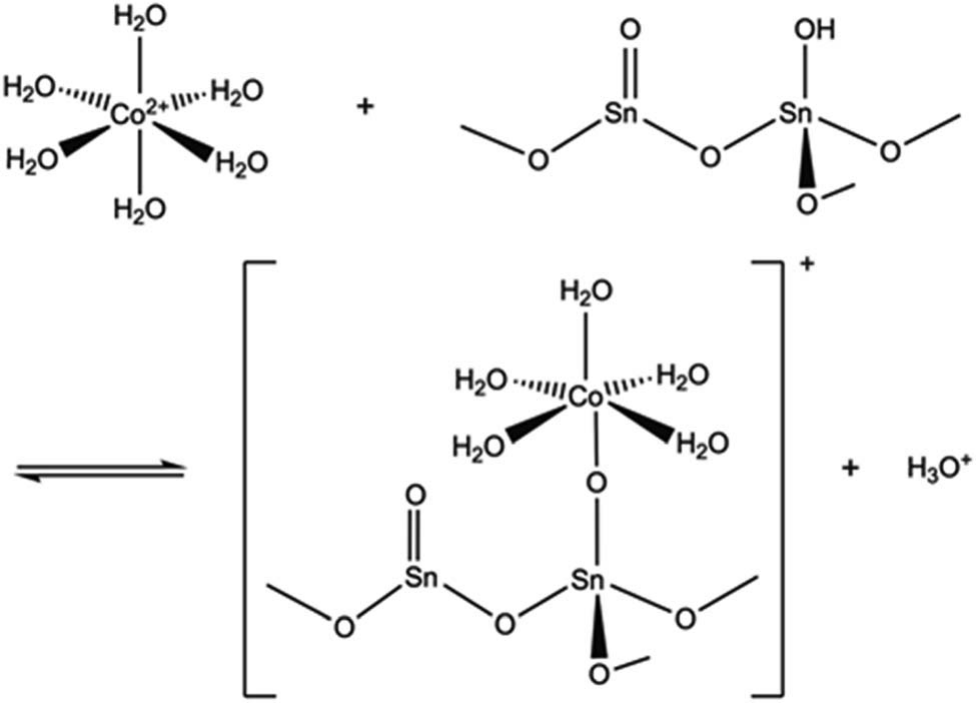
An assumed ion exchange reaction between Co^2+^ and a proton of surface hydroxyl group of SnO_2_.^5^

A good selectivity of the adsorbent is crucial when a trace amount of a specific ion is separated from a solution containing much higher concentrations of other ions. Thus, we examined how the Co^2+^ adsorption on SnO_2_ fibers is influenced by Na^+^ and Ca^2+^ ions that are among the most common cations in natural and nuclear waste waters with concentrations of 0.47 and 0.01 M in sea water, respectively.^67^ As Fig. 16 reveals, the Co^2+^ uptake remains high in the presence of Na^+^ ions irrespective of their concentration. By contrast, there is a marked weakening of the uptake in the CaCl_2_ solution at concentrations of 0.01 M or higher. With both of the ions, the interfering effect increases with concentration up to 0.1 M, after which there is no further decreasing effect on the uptake of Co^2+^. The good selectivity of the fibers for Co^2+^ over Na^+^ is promising for their use in decontamination of radioactive waste water, since Na^+^ is the most abundant coexisting ion in nuclear waste effluents.^67^

## 4 Conclusions

We have synthesized SnO_2_ and composite SnO_2_/SiO_2_ submicron fibers with a Sn : Si molar ratio of 3 : 1 and studied the ability of the fibers to remove Co^2+^ from an aqueous solution. For the synthesis, a novel and efficient electroblowing method was used. The as-electroblown fibers were calcined in air at 400, 450 and 500 °C with varying heating rates in order to produce the desired ceramic material and to investigate the effect of calcination temperature and heating rate on the structure and Co^2+^ uptake by the fibers. The bare SnO_2_ fibers had a granular structure in the tetragonal rutile phase with an average diameter of 560 nm. The SnO_2_/SiO_2_ composite fibers had a smooth core possibly comprising small SnO_2_ grains in a SiO_2_ matrix with large SnO_2_ grains dispersed on the core and the average diameter of the fibers was 580 nm. The morphology of the surface SnO_2_ grains of the composite fibers was dependent on the calcination temperature and heating rate. Irrespective of the calcination temperature, a heating rate of 1 °C min^−1^ yielded surface SnO_2_ grains that were roundish and *ca.* 30 nm in diameter. In the composite fibers calcined at 500 °C with a heating rate of 1 °C min^−1^ or at 400 °C with faster heating rates of 5 and 10 °C min^−1^ there were also some surface SnO_2_ grains that were irregularly shaped and 200 nm to 1 μm in diameter.

Both bare SnO_2_ and composite SnO_2_/SiO_2_ fibers had a high Co^2+^ uptake with SnO_2_ fibers exhibiting slightly more efficient Co^2+^ separation. Calcination temperature and heating rate affected the Co^2+^ uptake by the fibers. Among the SnO_2_ fibers, the fibers calcined at 500 °C performed the best. Among the composite fibers, the fibers calcined at 400 °C performed the best, and within them the fibers calcined with a heating rate of 10 °C min^−1^ were superior to the fibers calcined with heating rates of 1 and 5 °C min^−1^. The SnO_2_/SiO_2_ composite fibers were more elastic and durable and easier to handle than the somewhat brittle SnO_2_ fibers which makes them ideal for use in flow-through separation columns. Overall, on the basis of our results, the mechanical strength of SnO_2_ submicron fibers can be enhanced by adding a moderate amount of SiO_2_ without compromising their adsorption performance too much. This approach might also be extended to other ceramic submicron and nanofibers that require improved mechanical properties in various applications.

## Author contributions

J. P.: conceptualization, methodology, investigation, data curation, formal analysis, validation, visualization, writing – original draft. S. W.: investigation. S. L.: investigation, formal analysis. M. H.: formal analysis. M. V.: investigation, formal analysis. M. K.: investigation, formal analysis. T. H.: investigation, formal analysis. M. R.: funding acquisition, supervision, writing – review & editing. R. K.: funding acquisition, supervision, writing – review & editing.

## Conflicts of interest

There are no conflicts of interest to declare.

## Supplementary Material

RA-011-D1RA01559A-s001

RA-011-D1RA01559A-s002

## References

[cit1] Yang P. , The Chemistry of Nanostructured Materials, World Scientific, Singapore, 2003

[cit2] Clearfield A. , Inorganic Ion Exchange Materials, CRC Press, Boca Raton, Florida, 1982

[cit3] Abdelkader E., Nadjia L., Rose-Noelle V. (2016). Int. J. Ind. Chem..

[cit4] Donaldson J. D., Fuller M. J. (1968). J. Inorg. Nucl. Chem..

[cit5] Jaffrezic-Renault N. (1978). J. Inorg. Nucl. Chem..

[cit6] Misak N. Z., Shabana E. I., Mikhail E. M., Ghoneimy H. F. (1992). React. Polym..

[cit7] Rãutiu R., White D. A. (1996). Solvent Extr. Ion Exch..

[cit8] White D. A., Rãutiu R. (1997). Chem. Eng. J..

[cit9] Lagashetty A., Venkataraman A. (2004). Bull. Mater. Sci..

[cit10] Koivula R. and Harjula R., Presented in part at Waste management 2007 Conference, Tucson, Arizona, USA, 25 Feb. to 1 Mar., 2007

[cit11] Nilchi A., Shariati Dehaghan T., Rasouli Garmarodi S. (2013). Desalination.

[cit12] Yogesh Kumar K., Vinuth Raj T. N., Archana S., Benaka Prasad S. B., Olivera S., Muralidhara H. B. (2016). Journal of Water Process Engineering.

[cit13] Haq S., Rehman W., Waseem M., Rehman M., Shah K. H. (2020). J. Inorg. Organomet. Polym. Mater..

[cit14] Presley R. E., Munsee C. L., Park C.-H., Hong D., Wager J. F., Keszler D. A. (2004). J. Phys. D: Appl. Phys..

[cit15] Rizzato A. P., Santilli C. V., Pulcinelli S. H., Messaddeq Y., Hammer P. (2004). J. Sol-Gel Sci. Technol..

[cit16] Wang Y., Jiang X., Xia Y. (2003). J. Am. Chem. Soc..

[cit17] Pinna N., Neri G., Antonietti M., Niederberger M. (2004). Angew. Chem., Int. Ed..

[cit18] Wang Y., Ramos I., Santiago-Aviles J. J. (2007). IEEE Sens. J..

[cit19] Zhang Y., He X., Li J., Miao Z., Huang F. (2008). Sens. Actuators, B.

[cit20] Zhang Y., Li J., An G., He X. (2010). Sens. Actuators, B.

[cit21] Park J. Y., Asokan K., Choi S.-W., Kim S. S. (2011). Sens. Actuators, B.

[cit22] Ab Kadir R., Li Z., Sadek A. Z., Abdul Rani R., Zoolfakar A. S., Field M. R., Ou J. Z., Chrimes A. F., Kalantar-Zadeh K. (2014). J. Phys. Chem. C.

[cit23] Santos J. P., Fernández M. J., Fontecha J. L., Matatagui D., Sayago I., Horrillo M. C., Gracia I. (2014). Sensors.

[cit24] Liu Y., Yang P., Li J., Matras-Postolek K., Yue Y., Huang B. (2016). RSC Adv..

[cit25] Gunji S., Jukei M., Shimotsuma Y., Miura K., Suematsu K., Watanabe K., Shimanoe K. (2017). J. Mater. Chem. C.

[cit26] Yang Z., Du G., Feng C., Li S., Chen Z., Zhang P., Guo Z., Yu X., Chen G., Huang S., Liu H. (2010). Electrochim. Acta.

[cit27] Granger G. , Restoin C., Roy P., Jamier R., Rougier S., Duclere J.-R., Lecomte A., Dauliat R. and Blondy J.-M., Micro-Structured and Specialty Optical Fibres IV, 2015, vol. 9507, p. 95070J

[cit28] Shan H., Wang X., Shi F., Yan J., Yu J., Ding B. (2017). ACS Appl. Mater. Interfaces.

[cit29] Malik L. A., Bashir A., Qureashi A., Pandith A. H. (2019). Environ. Chem. Lett..

[cit30] Muthusaravanan S., Sivarajasekar N., Vivek J. S., Paramasivan T., Naushad M., Prakashmaran J., Gayathri V., Al-Duaij O. K. (2018). Environ. Chem. Lett..

[cit31] Anirudhan T. S., Shainy F., Deepa J. R. (2019). Chem. Ecol..

[cit32] Naushad M., ALOthman Z. A., Awual Md. R., Alam M. M., Eldesoky G. E. (2015). Ionics.

[cit33] Vijayaraghavan K., Rangabhashiyam S., Ashokkumar T., Arockiaraj J. (2016). Sep. Sci. Technol..

[cit34] Lönnrot S., Suorsa V., Paajanen J., Hatanpää T., Ritala M., Koivula R. (2019). RSC Adv..

[cit35] Ramakrishna S. , Fujihara K., Teo W.-E., Lim T.-C. and Ma Z., An Introduction to Electrospinning and Nanofibers, World Scientific, Singapore, 2005

[cit36] Thenmozhi S., Dharmaraj N., Kadirvelu K., Kim H. Y. (2017). Mater. Sci. Eng., B.

[cit37] Elmarco , https://elmarco.com/, accessed January 2021

[cit38] Fanavaran Nano-Meghyas (Fnm co. Ltd.) , http://en.fnm.ir/, accessed January 2021

[cit39] Inovenso Ltd. , https://www.inovenso.com/, accessed January 2021

[cit40] Daristotle J. L., Behrens A. M., Sandler A. D., Kofinas P. (2016). ACS Appl. Mater. Interfaces.

[cit41] Huang Z., Kolbasov A., Yuan Y., Cheng M., Xu Y., Rojaee R., Deivanayagam R., Foroozan T., Liu Y., Amine K., Lu J., Yarin A. L., Shahbazian-Yassar R. (2020). ACS Appl. Mater. Interfaces.

[cit42] Tutak W., Sarkar S., Lin-Gibson S., Farooque T. M., Jyotsnendu G., Wang D., Kohn J., Bolikal D., Simon Jr. C. G. (2013). Biomaterials.

[cit43] Bolbasov E. N., Stankevich K. S., Sudarev E. A., Bouznik V. M., Kudryavtseva V. L., Antonova L. V., Matveeva V. G., Anissimov Y. G., Tverdokhlebov S. I. (2016). Mater. Chem. Phys..

[cit44] Paajanen J., Lönnrot S., Heikkilä M., Meinander K., Kemell M., Hatanpää T., Ainassaari K., Ritala M., Koivula R. (2019). Nanoscale Adv..

[cit45] Um I. C., Fang D., Hsiao B. S., Okamoto A., Chu B. (2004). Biomacromolecules.

[cit46] Hsiao H.-Y., Huang C.-M., Liu Y.-Y., Kuo Y.-C., Chen H. (2012). J. Appl. Polym. Sci..

[cit47] WWW Table of Radioactive Isotopes , http://nucleardata.nuclear.lu.se/toi/nuclide.asp?iZA=270060, accessed January 2021

[cit48] Harjula R., Lehto J., Paajanen A., Brodkin L. (1999). presented in part at Waste Management'99 Conference, Tucson, Arizona. USA 28 Feb.–4 Mar.

[cit49] Holopainen J., Ritala M. (2016). J. Eur. Ceram. Soc..

[cit50] Santibenchakul S., Chaiyasith S., Pecharapa W. (2016). Integr. Ferroelectr..

[cit51] Reynolds A. S., Pierre T. H., McCall R., Wu J., Gato W. E. (2018). J. Environ. Sci. Health, Part A: Toxic/Hazard. Subst. Environ. Eng..

[cit52] Lutterotti L., Chateigner D., Ferrari S., Ricote J. (2004). Thin Solid Films.

[cit53] Santala E., Koivula R., Harjula R., Ritala M. (2019). Environ. Technol..

[cit54] Hsiao C. N., Huang K. S. (2005). J. Appl. Polym. Sci..

[cit55] Al-Harbi L. M., Kosa S. A., Baloch M. K., Bhatti Q. A., El-Mossalamy E. E. H. (2016). Int. J. Polym. Sci..

[cit56] Xia X., Dong X. J., Wei Q. F., Cai Y. B., Lu K. Y. (2012). eXPRESS Polym. Lett..

[cit57] Chiodini N., Meinardi F., Morazzoni F., Padovani J., Paleari A., Scotti R., Spinolo G. (2001). J. Mater. Chem..

[cit58] Denker B. I., Galagan B. I., Iskhakova L. D., Sverchkov S. E., Dianov E. M. (2015). Appl. Phys. B: Lasers Opt..

[cit59] Leonhardt C., Brumm S., Seifert A., Cox G., Lange A., Ruffer T., Schaarschmidt D., Lang H., Jöhrmann N., Hietschold M., Simon F., Mehring M. (2013). ChemPlusChem.

[cit60] Ohgi H., Maeda T., Hosono E., Fujihara S., Imai H. (2005). Cryst. Growth Des..

[cit61] Denry I., Holloway J. A., Gupta P. K. (2012). J. Biomed. Mater. Res., Part B.

[cit62] Mudra E., Shepa I., Milkovic O., Dankova Z., Kovalcikova A., Annušová A., Majkova E., Dusza J. (2019). Appl. Surf. Sci..

[cit63] Trujillano R., Villain F., Louis C., Lambert J.-F. (2007). J. Phys. Chem. C.

[cit64] Collins R. N., Kinsela A. S. (2010). Chemosphere.

[cit65] Koivula R., Harjula R., Lehto J. (2002). Microporous
Mesoporous Mater..

[cit66] Granados F., Bertin V., Bulbulian S., Solache-Ríos M. (2006). Appl. Radiat. Isot..

[cit67] Lehto J., Koivula R., Leinonen H., Tusa E., Harjula R. (2019). Sep. Purif. Rev..

