# Supplementary material for: Novel electroblowing synthesis of tin dioxide and composite tin dioxide/silicon dioxide submicron fibers for cobalt(ii) uptake
Source: RSC Adv. 2021 Apr 23;11(25):15245–57. doi: 10.1039/d1ra01559a (PMC8698243; doi:10.1039/d1ra01559a)
Supplement: RA-011-D1RA01559A-s002 [file RA-011-D1RA01559A-s002.pdf]

Electronic Supplementary Material (ESI) for RSC Advances.
This journal is © The Royal Society of Chemistry 2021

Supplementary Information

Novel Electroblowing Synthesis of Tin Dioxide and Composite Tin Dioxide/Silicon Dioxide Submicron Fibers for Cobalt(II) Uptake

Johanna Paajanen,*[a] Saara Weintraub,[a] Satu Lönnrot,[a] Mikko Heikkilä,[a] Marko Vehkamäki,[a] Marianna Kemell,[a] Timo Hatanpää,[a] Mikko Ritala[a] and Risto Koivula[a]

[a] *Department of Chemistry, P.O. Box 55, FI-00014 University of Helsinki, Finland*
E-mail: johanna.paajanen@helsinki.fi

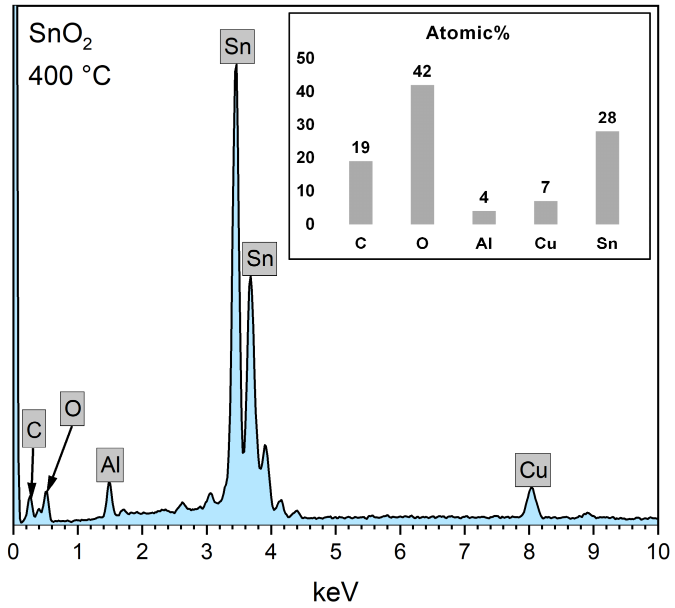

Fig. S1. EDX spectrum and elemental composition of the SnO_2 fibers calcined at 400 °C with a heating rate of 1 °C min^{-1}. In this and Fig. S2 to S4, carbon originates in the environment, glue in the adhesive tape and polymer residues, aluminium peaks are due to the material of the microscope and copper peaks are due to the tape used for sample preparation.

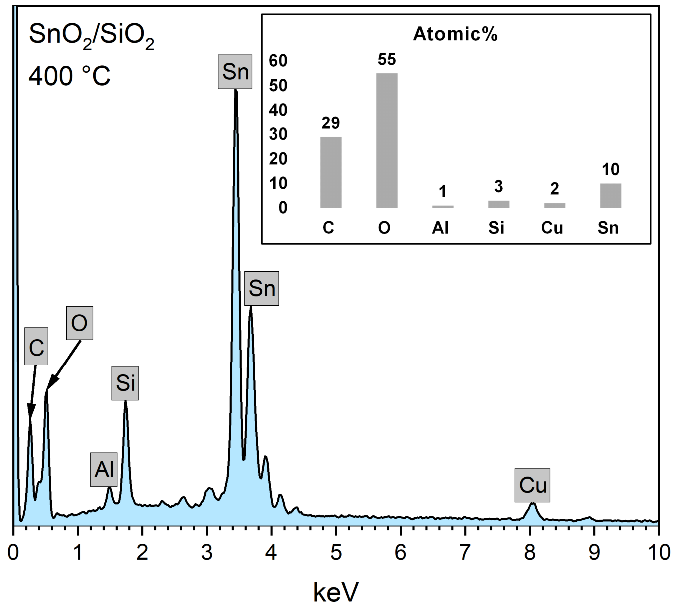

Fig. S2. EDX spectrum and elemental composition of the SnO_2/SiO_2 fibers calcined at 400 °C with a heating rate of 1 °C min^{-1}.

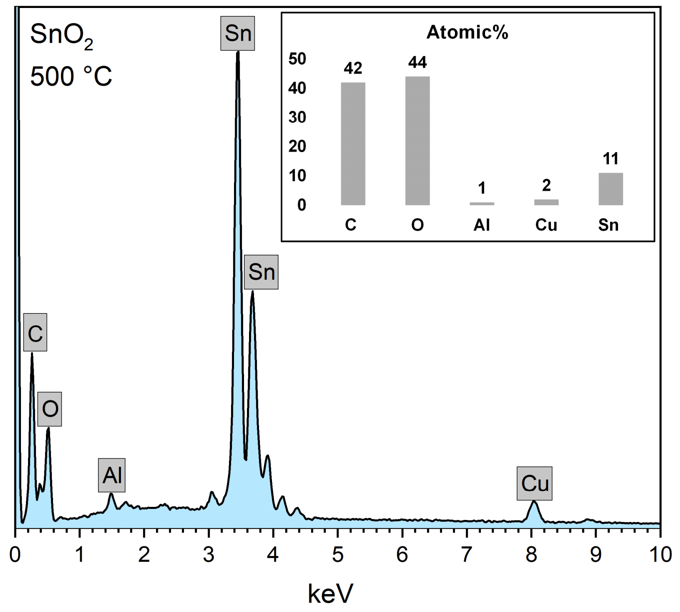

Fig. S3. EDX spectrum and elemental composition of the SnO_2 fibers calcined at 500 °C with a heating rate of 1 °C min^{-1}.

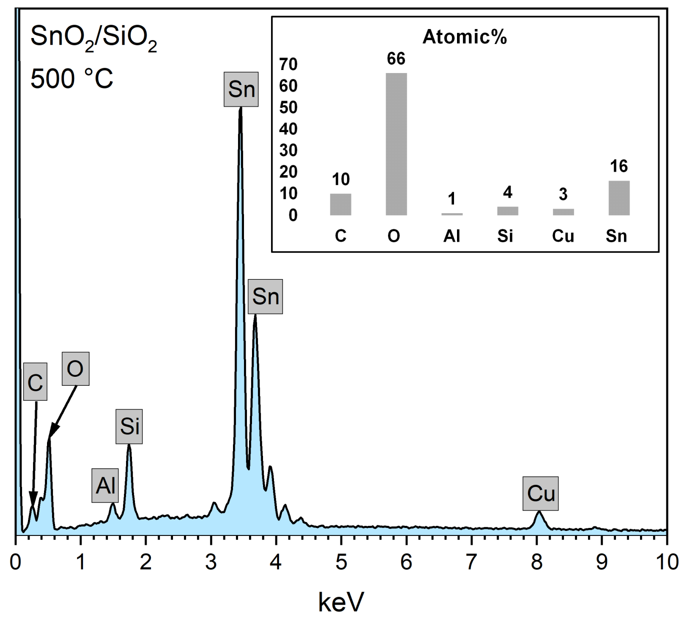

Fig. S4. EDX spectrum and elemental composition of the SnO_2/SiO_2 fibers calcined at 500 °C with a heating rate of 1 °C min^{-1}.

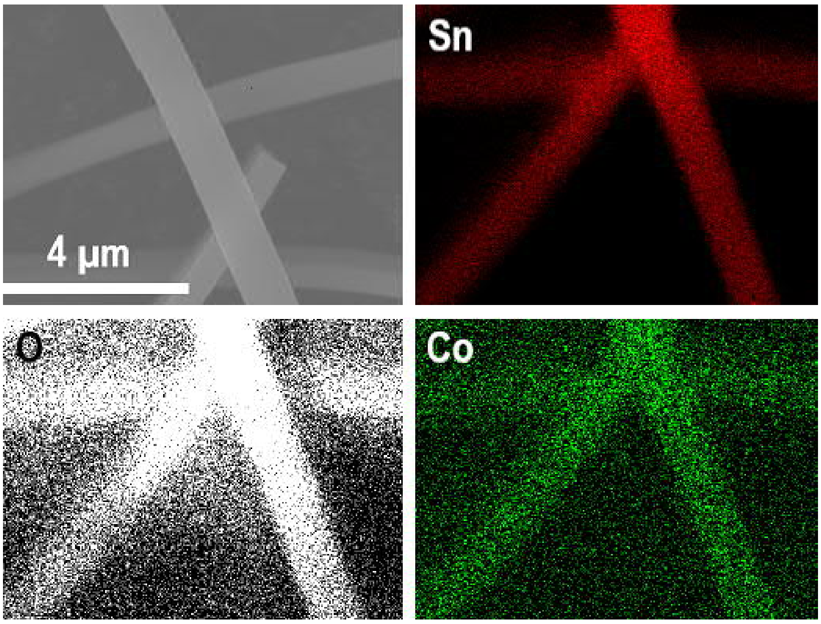

Fig. S5. EDX elemental maps of the SnO_2 fibers calcined at 400 °C with a heating rate of 1 °C min^{-1} after adsorption of Co^{2+}.

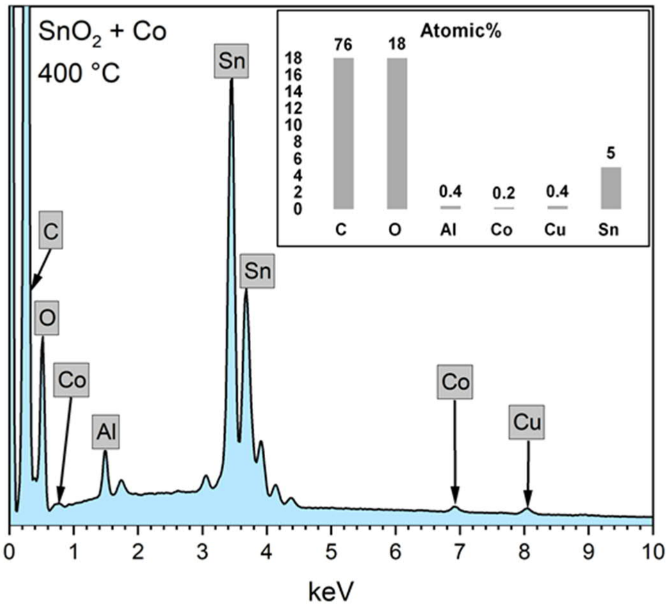

Fig. S6. EDX spectrum and elemental composition of the SnO_2 fibers calcined at 400 °C with a heating rate of 1 °C min^{-1} after adsorption of Co^{2+}. In this and Fig. S8, S10 and S12, carbon originates mainly in the tape used for sample preparation, aluminium peaks are due to the material of the microscope and the small copper peaks are probably due to the material of the sample holder.

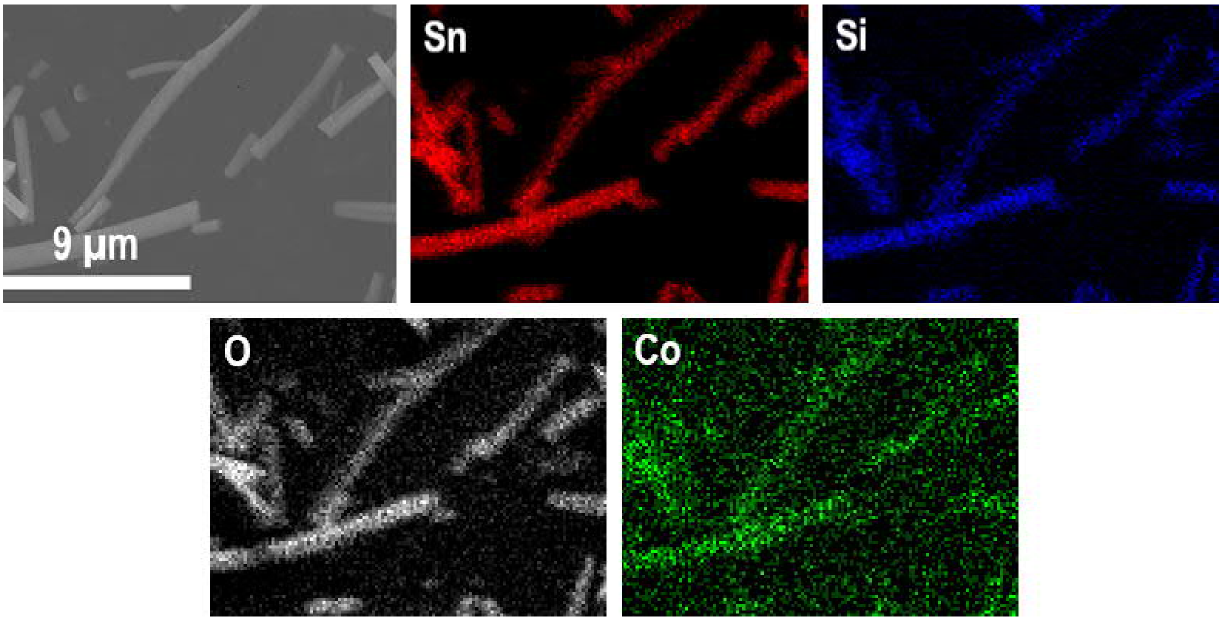

Fig. S7. EDX elemental maps of the SnO_2/SiO_2 fibers calcined at 400 °C with a heating rate of 1 °C min^{-1} after adsorption of Co^{2+}.

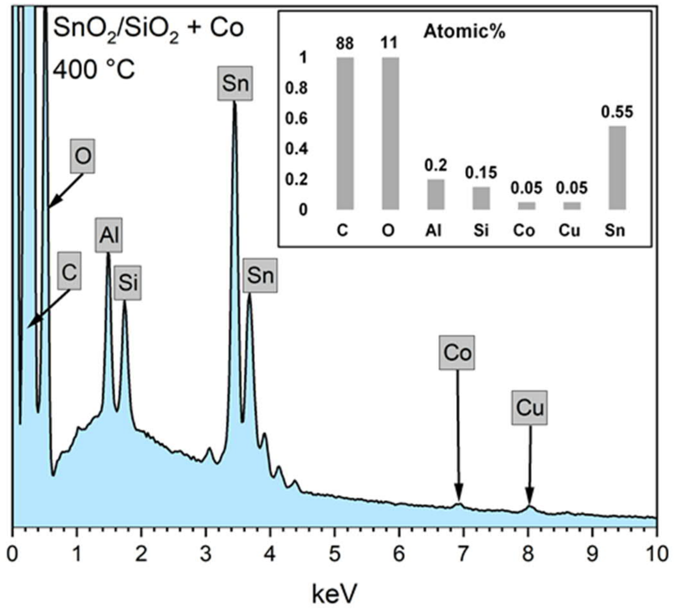

Fig. S8. EDX spectrum and elemental composition of the SnO_2/SiO_2 fibers calcined at 400 °C with a heating rate of 1 °C min^{-1} after adsorption of Co^{2+}.

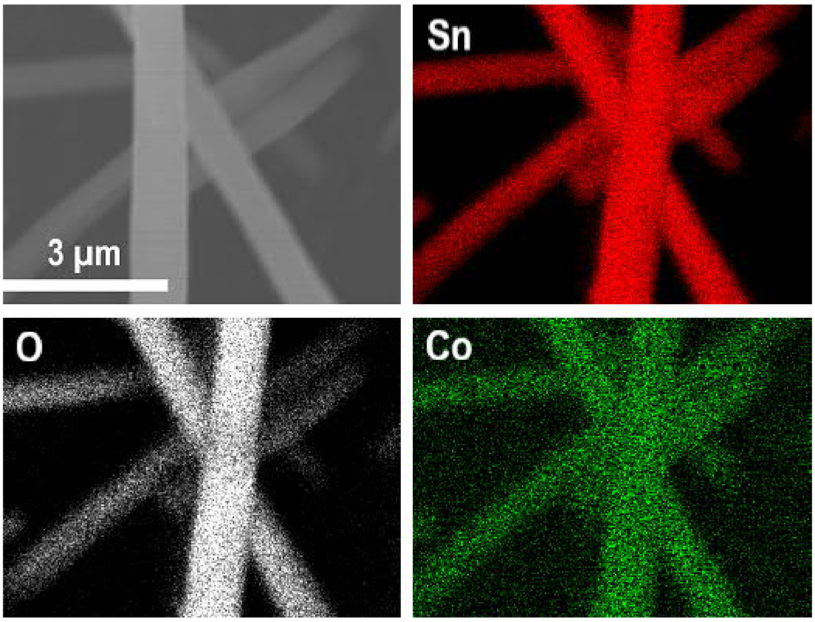

Fig. S9. EDX elemental maps of the SnO_2 fibers calcined at 500 °C with a heating rate of 1 °C min^{-1} after adsorption of Co^{2+}.

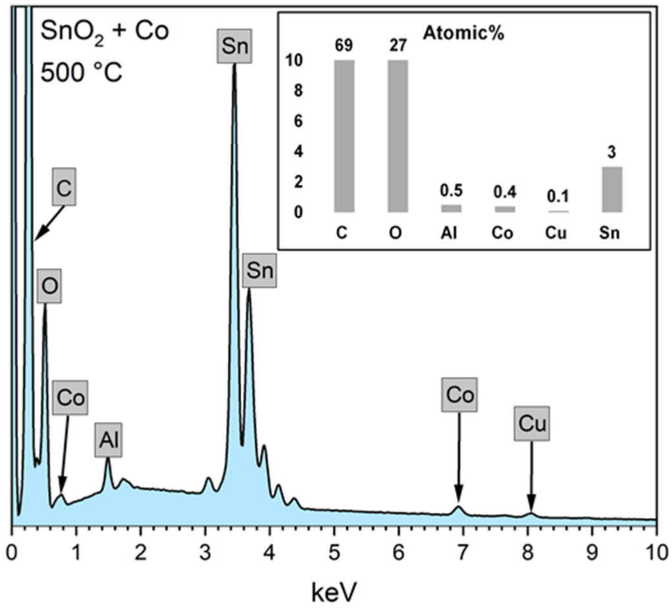

Fig. S10. EDX spectrum and elemental composition of the SnO_2 fibers calcined at 500 °C with a heating rate of 1 °C min^{-1} after adsorption of Co^{2+}.

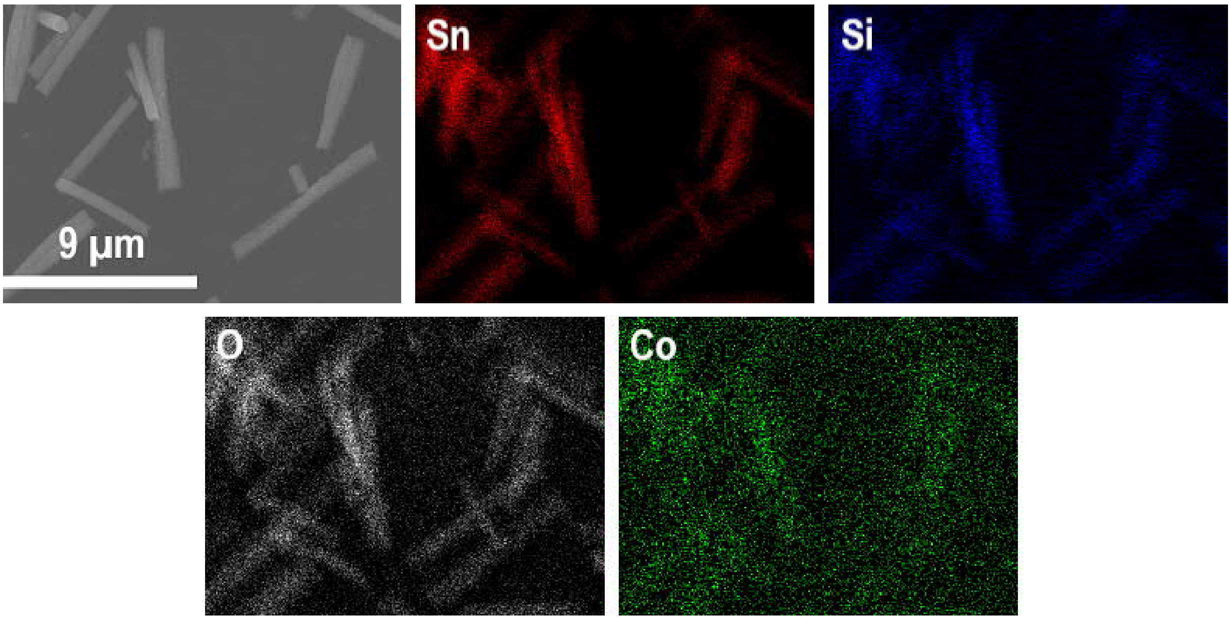

Fig. S11. EDX elemental maps of the SnO_2/SiO_2 fibers calcined at 500 °C with a heating rate of 1 °C min^{-1} after adsorption of Co^{2+}.

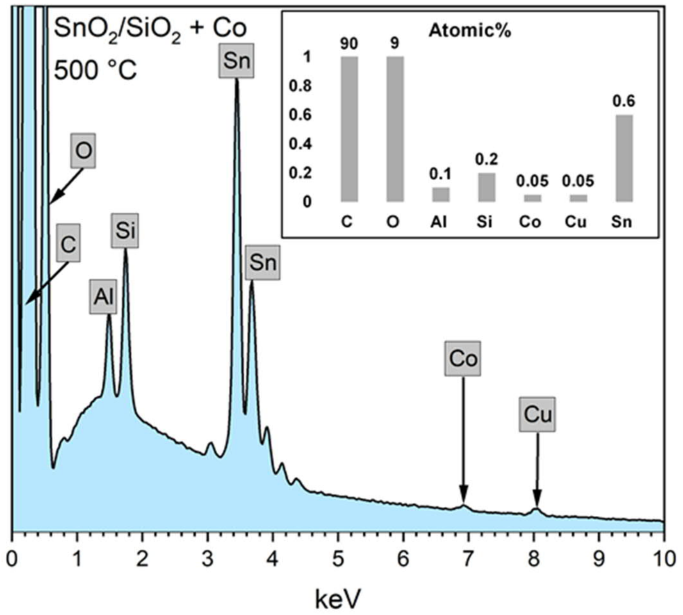

Fig. S12. EDX spectrum and elemental composition of the SnO_2/SiO_2 fibers calcined at 500 °C with a heating rate of 1 °C min^{-1} after adsorption of Co^{2+}.

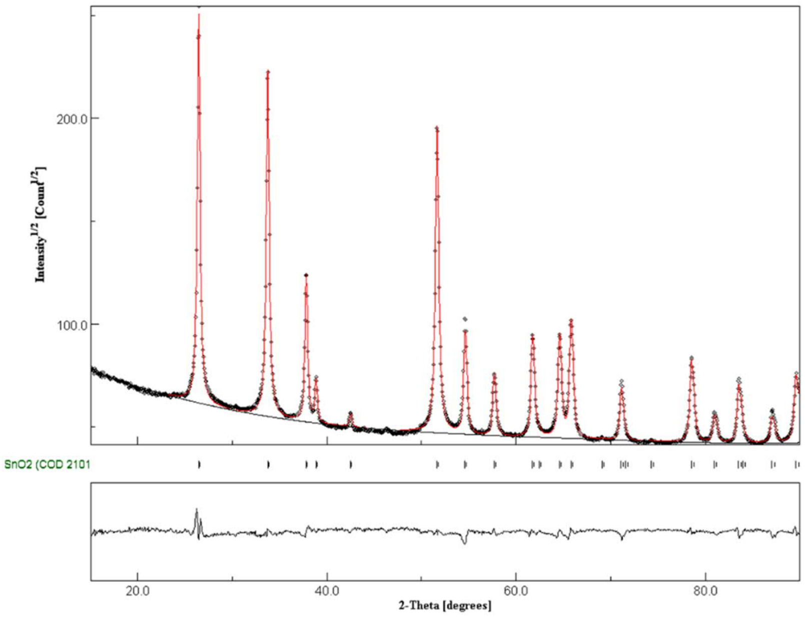

Fig. S13. Rietveld refined powder X-ray diffractogram of SnO_2 fibers calcined at 500 °C with a heating rate of 1 °C min^{-1}. In this and Fig. S14, the dots present the experimental data, the continuous lines present the calculated fit and the lowest curve shows the difference between experimental and calculated patterns.

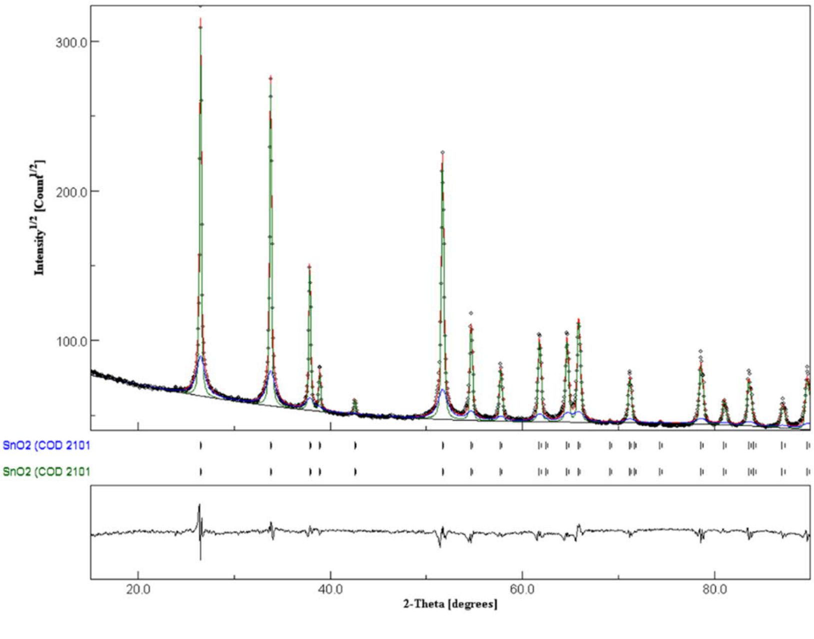

Fig. S14. Rietveld refined powder X-ray diffractogram of SnO_2/SiO_2 fibers calcined at 500 °C with a heating rate of 1 °C min^{-1}.

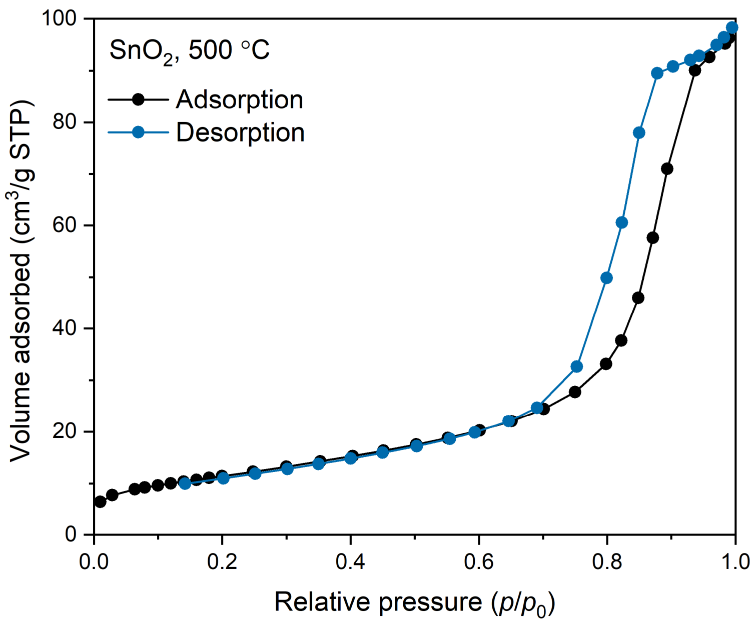

Fig. S15. N_2 adsorption and desorption isotherms of SnO_2 fibers calcined at 500 °C with a heating rate of 1 °C min^{-1}.

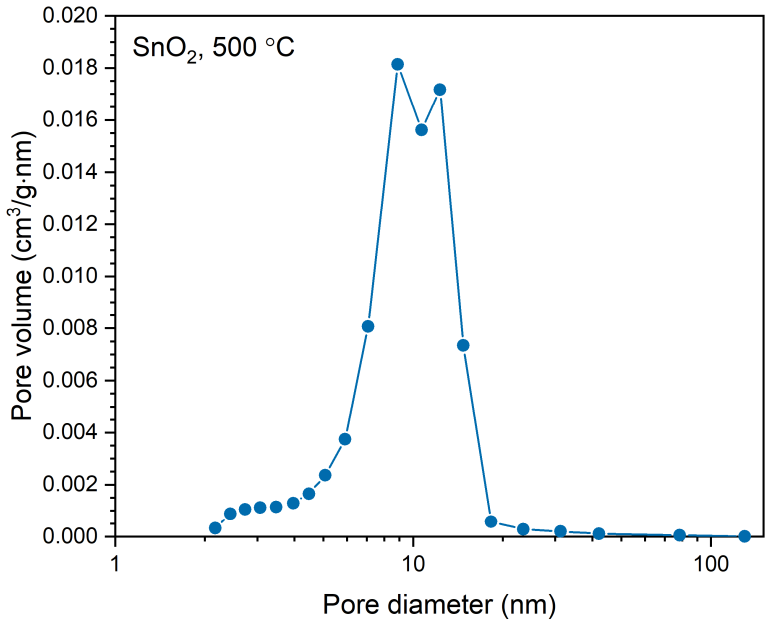

Fig. S16. Pore size distribution of SnO_2 fibers calcined at 500 °C with a heating rate of 1 °C min^{-1}.
